# A Vaccinomics Approach for the Identification of Tick Protective Antigens for the Control of *Ixodes ricinus* and *Dermacentor reticulatus* Infestations in Companion Animals

**DOI:** 10.3389/fphys.2019.00977

**Published:** 2019-07-26

**Authors:** Marinela Contreras, Margarita Villar, José de la Fuente

**Affiliations:** ^1^SaBio, Instituto de Investigación en Recursos Cinegéticos (IREC; CSIC-UCLM-JCCM), Ciudad Real, Spain; ^2^Department of Veterinary Pathobiology, Center for Veterinary Health Sciences, Oklahoma State University, Stillwater, OK, United States

**Keywords:** dog, rabbit, vaccine, tick, *Ixodes*, *Dermacentor*, transcriptomics, proteomics

## Abstract

Ticks and tick-borne pathogens affect health and welfare of companion animals worldwide, and some human tick-borne diseases are associated with exposure to domestic animals. Vaccines are the most environmentally friendly alternative to acaracides for the control of tick infestations, and to reduce the risk for tick-borne diseases affecting human and animal health. However, vaccines have not been developed or successfully implemented for most vector-borne diseases. The main limitation for the development of effective vaccines is the identification of protective antigens. To address this limitation, in this study we used an experimental approach combining vaccinomics based on transcriptomics and proteomics data with vaccination trials for the identification of tick protective antigens. The study was focused on *Ixodes ricinus* and *Dermacentor reticulatus* that infest humans, companion animals and other domestic and wild animals, and transmit disease-causing pathogens. Tick larvae and adult salivary glands were selected for analysis to target tick organs and developmental stages playing a key role during tick life cycle and pathogen infection and transmission. Two *I. ricinus* (heme lipoprotein and uncharacterized secreted protein) and five *D. reticulatus* (glypican-like protein, secreted protein involved in homophilic cell adhesion, sulfate/anion exchanger, signal peptidase complex subunit 3, and uncharacterized secreted protein) proteins were identified as the most effective protective antigens based on the criteria of vaccine *E* > 80%. The putative function of selected protective antigens, which are involved in different biological processes, resulted in vaccines affecting multiple tick developmental stages. These results suggested that the combination of some of these antigens might be considered to increase vaccine efficacy through antigen synergy for the control of tick infestations and potentially affecting pathogen infection and transmission. These antigens were proposed for commercial vaccine development for the control of tick infestations in companion animals, and potentially in other hosts for these tick species.

## Introduction

Tick-borne pathogens cause medically important infections affecting dogs and other pet species worldwide (Liu et al., 2018; Skotarczak, 2018), and some human tick-borne diseases are associated with exposure to domestic animals (Estrada-Peña et al., 2008; Kwit et al., 2018; Escárcega-Ávila et al., 2019). The tick species (Acari: Ixodidae), *Ixodes ricinus* (Linnaeus, 1758) and *Dermacentor reticulatus* (Fabricius, 1794) infest humans, pets and other domestic and wild animals. *I. ricinus* transmits disease-causing pathogens such as *Borrelia* spp. (Lyme disease and various borreliosis), tick-borne encephalitis virus (TBEV; tick-borne encephalitis) and *Anaplasma phagocytophilum* (human and animal anaplasmosis) while *D. reticulatus* is a vector for *Francisella tularensis* (tularemia), *Rickettsia* spp. (human and animal rickettsiosis), Omsk hemorrhagic fever virus (OHFV; Omsk hemorrhagic fever), and *Babesia canis* (canine babesiosis) (Glickman et al., 2006; de la Fuente et al., 2008, 2015; Beugnet and Marié, 2009).

Vaccines have not been developed or successfully implemented for most vector-borne diseases (VBD) affecting humans and animals (de la Fuente et al., 2017b). Therefore, reduction of arthropod vector infestations is important for the control of VBD (de la Fuente and Kocan, 2003; Sperança and Capurro, 2007; Karunamoorthi, 2011; Coller et al., 2012; de la Fuente and Contreras, 2015; de la Fuente et al., 2017b; de la Fuente, 2018). Traditional control methods for arthropod vector infestations are based on the use of chemical acaricides with associated drawbacks such as selection of arthropod-resistant strains and contamination of both the environment and animal products (de la Fuente and Kocan, 2003; de la Fuente et al., 2017b). Vaccination is an environmentally friendly alternative for the control of vector infestations and pathogen infections that allows control of several VBD by targeting their common vector (de la Fuente et al., 2007, 2011, 2017b; de la Fuente and Contreras, 2015; de la Fuente, 2018). Vaccines could be developed to target different tick developmental stages and functions on various hosts with the advantage of avoiding environmental contamination and selection of pesticide resistant arthropod vectors while improving animal welfare and production (de la Fuente et al., 2017b; de la Fuente, 2018). The experience with the only commercial vaccines available for the control of ectoparasite infestations, TickGard and Gavac, demonstrated that these vaccines contribute to the control of cattle tick populations while reducing acaricide applications, but were difficult to introduce into the market because of the absence of immediate effect on tick infestations and the application in combination with other control measures (de la Fuente and Kocan, 2003, 2006; Willadsen, 2004; de la Fuente et al., 2007). The hypothesis behind tick vaccine protective capacity is that ticks feeding on immunized hosts ingest antibodies specific for the target antigen that could reduce its levels and biological activity and/or interact with conserved epitopes in other proteins resulting in reduced tick feeding, development and reproduction (de la Fuente et al., 2011, 2017b; Moreno-Cid et al., 2011; de la Fuente, 2018).

The limiting step in developing tick vaccines is the identification of protective antigens (de la Fuente and Kocan, 2003; de la Fuente et al., 2018). Recent developments in omics analyses of both ticks and tick-borne pathogen and the application of systems biology to the study of tick-host-pathogen molecular interactions have advanced our understanding of the genetic factors and molecular pathways involved at the tick-host, tick-pathogen and host-pathogen interface (de la Fuente, 2012; de la Fuente et al., 2017a). These technologies are generating extensive information, but algorithms are needed to use these data for advancing knowledge on basic biological questions and the discovery of candidate protective antigens for the development of improved vaccines for the control of ticks and VBD (de la Fuente and Merino, 2013; de la Fuente et al., 2016, 2018; Contreras et al., 2018).

Acaricides have been effectively used to reduce tick infestations in companion animals (Otranto, 2018). However, ticks and transmitted pathogens continue to be a major health problem in dogs and other companion animals that require the development of effective vaccines (Otranto, 2018). Previous experiments have shown the possibility of using vaccines with tick gut protein extracts, Subolesin/Akirin and Bm96 for the control of *Rhipicephalus sanguineus* infestations in dogs (de la Fuente et al., 2015), and with pathogen-derived antigens for the control of VBD (e.g., Alhassan et al., 2018; Grosenbaugh et al., 2018). Nevertheless, vaccines for the control of tick infestations and transmitted diseases in companion animals have not been registered and commercialized.

In this study, we used a vaccinomics approach based on transcriptomics and proteomics data (de la Fuente and Merino, 2013; de la Fuente et al., 2016) in combination with vaccination trials for the discovery of tick protective antigens for the control of *I. ricinus* and *D. reticulatus* infestations in companion animals. Dogs and rabbits were used as model animals. Tick larvae and adult salivary glands were selected for analysis. Tick larvae are the first developmental stage to infest hosts and acquire infection or transmit pathogens that are transovarially transmitted. Salivary glands produce a cocktail of anti-clotting, anti-platelet, anti-inflammatory, vasodilatatory and immunomodulatory proteins that interfere with host defenses to aid in tick blood feeding and pathogen infection and transmission (Ayllón et al., 2015; Chmelaø et al., 2016; Nuttall, 2018). The experiments resulted in the identification of new antigens that showed a protective efficacy of vaccination against *I. ricinus* and *D. reticulatus* infestations in rabbits and dogs.

## Materials and Methods

### Ethics Statement

White rabbits (*Oryctolagus cuniculus*) and Beagle dogs (*Canis lupus familiaris*) were used in the experiments. Animal experiments were conducted in strict accordance with the recommendations of the European Guide for the Care and Use of Laboratory Animals. Animals were housed and experiments conducted at LLC ACRO Vet Lab (Pylipovichi village, Kiev region, Ukraine) with the approval and supervision of the Ukrainian Commission for Bioethics and Biosafety for animals under the studies “Tick vaccine experiment on rabbits” number 000369 and “Tick vaccine experiments on dogs” numbers 000576, 000577 and 000761.

### Ticks and Sample Preparation

*Ixodes ricinus* ticks were originally obtained from the reference laboratory colony maintained at the tick rearing facility of the Institute of Parasitology of the Biology Centre of the Academy of Sciences of the Czech Republic (Genomic Resources Development Consortium, Contreras et al., 2014). *D. reticulatus* ticks were obtained from a Dutch colony maintained at the Utrecht Centre for Tick-borne Diseases (UCTD), Department of Infectious Diseases and Immunology, Faculty of Veterinary Medicine, Utrecht University, Utrecht, The Netherlands (Villar et al., 2014). Tick colonies were maintained at the LLC ACRO Vet Lab (Pylipovichi village, Kiev region, Ukraine) and used for analysis and vaccination trials. *I. ricinus* unfed larvae whole internal tissues (IL) and unfed adult salivary glands (ISG), and *D. reticulatus* unfed larvae whole internal tissues (DL) and unfed adult salivary glands (DSG) were processed. RNA and proteins were extracted from approximately 500 larvae using the AllPrep DNA/RNA/Protein Mini Kit (Qiagen, Valencia, CA, United States) and from 50 salivary glands (25 females and 25 males) using Tri Reagent (Sigma-Aldrich, St. Louis, MO, United States) according to manufacturer instructions. RNA was purified with the RNeasy MinElute Cleanup Kit (Qiagen, Valencia, CA, United States) and characterized using the Agilent 2100 Bioanalyzer (Santa Clara, CA, United States) in order to evaluate the quality and integrity of RNA preparations. RNA concentration was determined using the Nanodrop ND-1000 (NanoDrop Technologies Wilmington, DE, United States). Protein concentration was determined using the Pierce BCA Protein Assay Kit (Thermo Scientific, Rockford, IL, United States) with BSA as standard. RNA yield was 10 μg (IL), 4 μg (ISG), 3.5 μg (DL), and 6 μg (DSG). Protein yield was 8 mg (IL and DL), 1.4 mg (ISG), and 3 mg (DSG).

### Transcriptomics Data Acquisition for *I. ricinus* and *D. reticulatus* Samples

Purified RNAs were used for library preparation using the TruSeq RNA sample preparation kit v.1 and the standard low throughput procedure (Illumina, San Diego, CA, United States) as previously reported (Genomic Resources Development Consortium, Contreras et al., 2014; Villar et al., 2014). Briefly, 1 μg total RNA was used as starting material for library preparation with the exception of the DL sample for which 0.7 μg were used. Messenger RNA was captured using poly-dT magnetic beads and purified polyA+ RNA was chemically fragmented and reverse-transcribed. Remaining RNA was enzymatically removed, and the second strand generated following an end repair procedure and preparation of double-stranded cDNA for adaptor ligation. Adaptor oligonucleotides containing the signals for subsequent amplification and sequencing were ligated to both ends and cDNA samples were washed using AMPure SPRI-based magnetic beads (Beckman Coulter, IZASA, Barcelona, Spain). Adapters contained identifiers, which allow multiplexing in the sequencing run. An enrichment procedure based on PCR was then performed to ensure that all molecules in the library conserved the adapters at both ends. The number of PCR cycles was adjusted to 10 for all samples except for DL which needed up to 15 cycles. The final amplified library was checked again on a BioAnalyzer 2100 (Agilent, Santa Clara, CA, United States). Libraries were titrated by quantitative PCR using a reference standard to characterize molecule concentration per library (IL, 5.51 nM; ISG, 62.16 nM; DL, 12.44 nM; DSG, 100.95 nM). Libraries were denatured and seeded on the respective lanes of the flowcells at a final concentration after re-naturalization of 10–14 pM. IL and ISG samples were run in single-nucleotide flowcells while DL and DSG samples were run in double-nucleotide flowcells to allow for pair-end sequencing. After binding, clusters were formed in the flowcells by local amplification using a Cluster Station apparatus (Illumina). Following sequencing primer annealing, flowcells were loaded into a GAIIx equipment (Illumina) to perform sequencing using the TruSeq^®^ system (Illumina). IL and ISG samples were run under a 1 × 75 bp single-end read protocol while DL and DSG samples were run under a pair-end 2 × 100 bp protocol for *de novo* RNA sequencing (RNAseq). After sequencing and quality filtering, reads were split according to their different identifiers and fastq files were generated to proceed to quality analysis and gene expression analysis and/or *de novo* transcript assembly. Transcriptomics data was submitted to Dryad Digital Repository^1^ or published here (Supplementary Dataset 1) and by Villar et al. (2014) for *I. ricinus* and *D. reticulatus* samples, respectively.

### Bioinformatics for *I. ricinus* and *D. reticulatus* Transcriptomics Data

#### *I. ricinu*s

The pipeline used for bioinformatics analysis of *I. ricinus* transcriptomics data was similar to that described by Twine et al. (2011) as previously reported (Genomic Resources Development Consortium, Contreras et al., 2014). The reads from both IL and ISG samples were mapped to the reference *I. scapularis* genome (assembly JCVI_ISG_i3_1.0^2^) using a TopHat-Cufflinks-Cuffdiff pipeline (Langmead et al., 2009; Trapnell et al., 2009). Transcript abundance was estimated using Cufflinks by analyzing the number of reads mapped to each transcript and handling the deviations due to the sample preparation (Langmead et al., 2009). Finally, differential expression between IL and ISG was characterized using Cuffdiff, integrated into the Cufflinks package (Langmead et al., 2009). For analysis of genes coding for secreted proteins, 6 subcellular localization predictors were chosen: WoLF PSORT^3^, TargetP^4^, Protein Prowler^5^, SLP-Local^6^, PredSL^7^ and CELLO^8^. All these tools have web services that enable batch protein sequence submissions. Results for genes encoding for predicted secreted proteins in IL and ISG were included in Supplementary Dataset 2.

#### *D. reticulatu*s

For *D. reticulatus* transcriptomics data, the 21,838,507 reads (∼2.2 Gb) in DSG were compared against the *I. ricinus* selected reference gene set (Table 1) using PROmer (Kurtz et al., 2004). The query sequences were considered in their 3 reading frames and the reference sequences only in the frame 1. PROmer results were filtered by selecting the hits with a minimum length of 60 bp for the extended similarity region and a minimum identity of 80% in this region. Reads with hits fulfilling these requirements were clustered to the selected gene with which they have PROmer hits. We selected the read with hits and always also its paired read. The reads belonging to each cluster were then assembled with Velvet (Zerbino and Birney, 2008; Zerbino et al., 2009) to produce a set of contigs clustered to the reference genes. A BLASTX was then done against all proteins from Ixodidae included in the nr database. For each cluster of contigs, we chose the most similar protein to which the contigs aligned with more similarity, and substituted that protein as the new reference protein for that cluster. As expected, in many cases the most similar protein was identical to the initial *I. ricinus* reference protein. Differential gene expression between DL and DSG samples was determined using χ^2^ test statistics with Bonferroni correction (*P* = 0.05) in the IDEG6 software^9^.

**TABLE 1:** Selected *I. ricinus* genes encoding for candidate tick protective antigens.

***Ixodes* gene ID**	**Genbank accession No. for *I. scapularis***	**Genbank accession No. for *I. ricinus***	**Encoded protein**	**Expression profile**	**Selection criteria**
472	ISCW000027 XM_002433472	GFVZ01075347	Uncharacterized protein	Over in IL	Transcriptomics: Top 1,000 expressed genes in both IL and ISG encoding for putative secreted proteins
529^a^	ISCW002303 XM_002399529	GFVZ01179036	Uncharacterized protein	Over in IL	
752^b^	ISCW002070 XM_002403752	GADI01007371	DNA-bridging protein BAF	Over in IL	
082^c^	ISCW024295 XM_002434082	GFVZ01108982	Secreted protein	Over in IL	
869^a^	ISCW006458 XM_002434869	GFVZ01137642	Uncharacterized protein	Over in IL	
965^a^	ISCW023699 XM_002415965	GFVZ01166334	Conserved hypothetical protein	Over in IL	
922^a,c^	ISCW021228 XM_002403922	GEGO01004220	Vitellogenin 2	Over in IL	Proteomics: Search against Ixodidae database with FDR = 0.01, identification with more than 4 peptides and filtered for BP and redundancy
391^c^	ISCW021710 XM_002411391	GANP01014013	Heme lipoprotein	Over in IL	
749^c^	ISCW016308 XM_002407749	GFVZ01132208	Conserved hypothetical protein	Over in IL	
216^c^	ISCW010436 XM_002406216	GFVZ01099159	Secreted salivary gland peptide	Over in IL	Proteomics: Search against putative secreted proteins encoded by top 1,000 expressed genes in both IL and ISG with XCorr > 2 and sequence span > 2%
892^a^	ISCW002948 XM_002409892	GFVZ01048464	Gap1	Over in IL	
450^a^	ISCW018653 XM_002434450	GFVZ01135723	Vacuolar H+ ATPase	Over in IL	
950^a^	ISCW013911 XM_002415950	GFVZ01068232	Hypothetical protein	Over in IL	
158^c^	ISCW019448 XM_002401158	GEGO01005185	Glypican	Over in IL	
459	ISCW011801 XM_002411459	GFVZ01157992	Hypothetical protein	Over in IL	
251^a^	ISCW023637 XM_002416251	GADI01004860	Secreted metalloprotease	Over in ISG	
178^b^	ISCW017062 XM_002409178	GANP01001334	Zinc finger protein, Palmitoyltransferase	Over in ISG	
623	ISCW015091 XM_002414623	GFVZ01042726	Hypothetical protein	Over in ISG	
738^a^	ISCW012232 XM_002413738	GEFM01006608	Sodium/glucose cotransporter	Over in ISG	
874^a^	ISCW006195 XM_002399874	GFVZ01065454	Fasciclin domain-containing protein	Over in ISG	
908^a^	ISCW014557 XM_002414908	GFVZ01100779	Secreted protein	Over in ISG	
150	ISCW006313 XM_002436150	GANP01003227	Secreted protein	Over in ISG	
556^a^	ISCW006654 XM_002400556	GADI01006770	Gamma-interferon inducible lysosomal thiol reductase	Over in ISG	
016^c^	ISCW019023 XM_002436016	GFVZ01163301	Secreted protein	Over in ISG	
971^a^	ISCW006734 XM_002435971	GADI01005462	Hypothetical protein	Over in ISG	
964^a,c^	ISCW001819 XM_002402964	GFVZ01127380	Conserved hypothetical protein	Over in ISG	Transcriptomics: Biological process (BP) GO for genes predicted as encoding for secreted proteins that were among the top 1,000 expressed genes overexpressed in ISG and filtered for BP and redundancy
427^a^	ISCW015065 XM_002414427	GANP01009473	Solute carrier	Over in ISG	
490^c^	ISCW003957 XM_002399490	GANP01008639	Aquaporin	Over in ISG	
058^c^	ISCW023239 XM_002415058	GANP01004202	Sulfate/anion exchanger	Over in ISG	
912	ISCW002249 XM_002410912	GANP01011997	Sialin	Over in ISG	
078^a^	ISCW005630 XM_002435078	GFVZ01037382	Sialin	Over in ISG	
432	ISCW019986 XM_002404432	GANP01006518	Monocarboxylate transporter	Over in ISG	
098	ISCW013157 XM_002413098	GANP01011927	Sodium-dependent multivitamin transporter	Over in ISG	
624	ISCW015092 XM_002414624	GFVZ01042726	Sodium/solute symporter	Over in SG	
145^a^	ISCW019568 XM_002405145	GFVZ01171589	Signal peptidase 12 kDa subunit	IL = ISG	Transcriptomics: Biological process (BP) GO for genes predicted as encoding for secreted proteins that were among the top 1,000 expressed genes in both IL and ISG and filtered for BP and redundancy
256^a^	ISCW016779 XM_002403256	GFVZ01154332	Signal peptidase complex subunit 3	IL = ISG	

*^*a*^Genes abandoned due to no amplification by RT-PCR or low expression levels in *E. coli*. ^*b*^Genes removed from further analysis due to the GO CC prediction. ^*c*^Proteins identified in the membrane fraction of IL with FDR < 0.01. IL, *I. ricinus* larvae and ISG, salivary glands.*

### Proteomics Data Acquisition for *I. ricinus* and *D. reticulatus* Samples

Proteins were resuspended in 5% SDS and frozen at −20°C until use. Two hundred μg of protein extracts from the four samples, IL, ISG, DL and DSG, were mixed with the same quantity of Laemmli sample buffer x2 and applied using a 5-well comb on a conventional SDS-PAGE gel (1.5 mm-thick, 4% stacking, 10% resolving). The electrophoretic run was stopped as soon as the front entered 3 mm into the resolving gel, so that the whole proteome became concentrated in the stacking/resolving gel interface. The unseparated protein bands were visualized by Coomassie Brilliant Blue R-250 staining, excised, cut into cubes (2 × 2 mm) and treated with 0.1% RapiGest SF surfactant (Waters, Milford. MA, United States) according to manufacturer’s protocol in order to enhance the subsequent in-gel trypsin digestion. After RapiGest treatment, samples were digested overnight at 37°C with 60 ng/μl trypsin (Promega, Madison, WI, United States) at 5:1 protein:trypsin (w/w) ratio in 50 mM ammonium bicarbonate, pH 8.8 containing 10% (v/v) acetonitrile. The resulting tryptic peptides from each proteome were extracted by 1 h-incubation in 12 mM ammonium bicarbonate, pH 8.8. Trifluoroacetic acid (TFA) was added to a final concentration of 1% and the peptides were finally desalted onto C18 OASIS HLB extraction cartridges (Waters, Milford, MA, United States) and dried-down prior to reverse phase high performance liquid chromatography (RP-HPLC) method coupled with mass spectrometry (RP-HPLC-MS/MS) analysis using a Surveyor LC system coupled to an ion trap mass spectrometer model LCQ Fleet (Thermo-Finnigan, San Jose, CA, United States). Peptides were eluted using a 180-min gradient from 5 to 40% solvent B in solvent A (solvent A: 0.1% formic acid in water; solvent B: 0.1% formic acid in acetonitrile). The LCQ Fleet was programmed to perform a data-dependent MS/MS scan on the 3 most intense precursors detected in a full scan from 400 to 1600 amu (1 μscan, 200 ms injection time). Protein identification was conducted using SEQUEST algorithm (Proteome Discoverer 1.1 package, Thermo Finnigan), allowing optional modifications in Methionine (oxidation) and Cysteine (carboxamidomethylation). Proteomics data was included in Supplementary Dataset 3 or published by Villar et al. (2014) for *I. ricinus* and *D. reticulatus* samples, respectively.

### Bioinformatics for *I. ricinus* and *D. reticulatus* Proteomics Data

The MS/MS raw files were searched against the Ixodidae Uniprot database (28,771 entries in February 2012) with the following constraints: tryptic cleavage after Arginine and Lysine, up to two missed cleavage sites, and tolerances of 0.5 Da for precursor ions and 0.8 Da for MS/MS fragment ions. Protein assignations were first filtered with a SEQUEST XCorr > 2, but only proteins with false discovery rate (FDR) ≤ 0.01 and identified with at least 4 peptides were selected for further analysis from the Ixodidae database. The MS/MS data was also used to search against the database of predicted secreted proteins encoded by the selected top 1,000 expressed genes in both IL and ISG. In this case, proteins with sequence span > 2% were selected for further analysis. Differential protein representation between L and SG samples was determined using χ^2^ test statistics with Bonferroni correction (*P* = 0.001) in the IDEG6 software (see footnote 9).

### Gene Ontology

Gene ontology (GO) analysis for Molecular Function (MF), Cellular Component (CC) and/or Biological Process (BP) was conducted using the cDNA Annotation System software (dCAS; Bioinformatics and Scientific IT Program (BSIP), Office of Technology Information Systems (OTIS), National Institute of Allergy and Infectious Diseases (NIAID), Bethesda, MD, United States^10^), AmiGO^11^ and UniProt^12^ using non-redundant sequence database (nr) and databases of tick-specific sequences^13^, ^14^. The GO annotations for selected candidate tick protective antigens were included in Supplementary Dataset 4.

### Cloning of *I. ricinus* and *D. reticulatus* Genes Encoding for Candidate Protective Antigens

First, selected *I. ricinus* orthologs and *D. reticulatus de novo* generated contigs were amplified by RT-PCR with *I. scapularis* or *D. reticulatus* sequence-specific primers and conditions (Supplementary Table 1) using IL, ISG, DL and DSG RNA and the iScript One-Step RT-PCR Kit with SYBR Green and the iQ5 thermal cycler (Bio-Rad, Hercules, CA, United States) following manufacturer’s recommendations. The genes that did not produce an amplicon after RT-PCR were eliminated from further analyses (Tables 1, 2). Remaining coding regions for *I. scapularis* sequences corresponding to selected *I. ricinus* orthologous sequences and *D. reticulatus de novo* generated contig sequences were synthesized (GenScript, Hong Kong) with optimized codon usage for *Escherichia coli* (Supplementary Dataset 5).

**TABLE 2:** Selected *D. reticulatus* genes encoding for candidate tick protective antigens.

***Dermacentor* gene ID**	***Ixodes* gene ID**	**Genbank accession No. for *D. reticulatus***	**Number of reads**		**Expression profile (overexpressed in)**	
			
			**DL**	**DSG**	***I. ricinus***	***D. reticulatus***
S1^a,c^	964	MK895447	250	3206	ISG^*^	DSG^*^
S2	256	MK895448	881	1748	ISG^*^	DSG^*^
S3^b^	752	NA	68	82	IL	DSG
S4^b^	178	NA	35	181	ISG^*^	DSG^*^
S5	892	MK895449	4	216	IL	DSG
S6	912	MK895450	5	49	ISG^*^	DSG^*^
S7^c^	490	MK895451	45	2000	ISG^*^	DSG^*^
S8^c^	158	MK895452	0	34	IL	DSG
S9	145	MK895453	9	49	ISG^*^	DSG^*^
S10^c^	391	MK895454	0	240	IL	DSG
S11	459	MK895455	145	796	IL	DSG
S12	098	MK895456	84	246	NS	DSG
S13	738	MK895457	12	136	ISG^*^	DSG^*^
S14^c^	216	MK895458	3585	2088	IL^*^	DL^*^
S15	427	MK895459	30	702	ISG^*^	DSG^*^
S16	624	MK895460	49	481	NS	DSG
S17^c^	908	MK895461	867	3972	ISG^*^	DSG^*^
S18^c^	058	MK895462	23	749	ISG^*^	DSG^*^
S19	950	MK895463	2	14	IL	DSG
S20^c^	082	MK895464	67800	1839	IL^*^	DL^*^
S21	450	MK895465	64	105	IL	DSG
S22	078	MK895466	5	27	ISG^*^	DSG^*^
S23^c^	016	MK895467	9	139	ISG^*^	DSG^*^
S24^a^	150	NA	0	1	ISG^*^	DSG^*^
S25	874	MK895468	2	39	ISG^*^	DSG^*^
S26^a^	529	NA	2	0	IL	NS
S27^a^	749	NA	3	0	IL	NS

^*^Similar gene expression profile in *I. ricinus* and *D. reticulatus*. ^*a*^Genes abandoned due to poor sequence information or low expression in *E. coli*. ^*b*^Genes removed from further analysis due to the GO CC prediction. ^*c*^Proteins identified in the membrane fraction of DL with FDR < 0.01. NA, no accession number because sequences were not submitted due to poor sequence information or because genes were removed from further analysis due to the GO CC prediction. NS, not significant; IL, *I. ricinus* larvae and ISG, salivary glands; DL, *D. reticulatus* larvae and DSG, salivary glands.

### Production of Recombinant Proteins and Vaccine Formulations

For gene expression and the production of the recombinant proteins, *E. coli* BL21 Star (DE3) One Shot cells (Invitrogen-Life Technolgies, Inc., Grand Island, NY, United States) were transformed with the target gene cloned into the pET101/D-TOPO expression vector (Invitrogen-Life Technolgies). Recombinant *E. coli* were inoculated into 10 ml of Luria-Bertani (LB) containing 50 μg/ml ampicillin (Sigma-Aldrich, St Louis, MO, United States) and 0.4% glucose (Laboratorios CONDA S.A., Madrid, Spain) and kept growing overnight at 37°C with shaking. Two ml of the overnight culture was propagated into 250 ml flasks containing 50 ml LB, 50 μg/ml ampicillin and 0.4% glucose for 2 h at 37°C and 200 rpm, and then for 4 h after addition of 0.5 mM final concentration of isopropyl-β-d-thiogalactopyranoside (IPTG, Sigma-Aldrich) for induction of gene expression. The cells were harvested by centrifugation at 3,900 × *g* for 15 min at 4°C and stored at −80°C for protein purification. One g of the cells harvested after induction of gene expression were resuspended in 5 ml of lysis buffer (100 mM Tris-HCl, pH 7.5, 250 mM NaCl, 7 M Urea, 10 mM imidazole) containing protease inhibitor (Ref. 04693132001, Roche, San Cugat del Vallés, Barcelona, Spain) and disrupted using a cell sonicator (Model MS73; Bandelin Sonopuls, Berlin, Germany). After disruption, the insoluble protein fraction containing the recombinant antigens as inclusion bodies were collected by centrifugation at 15,000 × *g* for 15 min at 4°C and stored at −20°C before purification. The purification of the recombinant protein was conducted using the automated Maxwell 16 Polyhistidine Protein Purification Kit (Promega, Madison, WI, United States). The eluted fraction containing the purified denatured proteins was dialyzed against 1000 volumes of PBS (137 mM NaCl, 2.7 mM KCl, 10 mM Na_2_HPO_4_, 1.8 mM KH_2_PO_4_), pH 7.4 for 12 h at 4°C. Recombinant proteins were then concentrated using an Amicon Ultra-15 ultrafiltration device (cut off 10 kDa) (Millipore-Merck, Darmstadt, Germany), and adjusted to 0.5 mg/ml. Protein concentration was determined using bicinchoninic acid (Pierce BCA Protein Assay Kit, Thermo Scientific, Rockford, IL, United States). For vaccine formulation, recombinant proteins or saline control were adjuvated in Montanide ISA 50 V2 (Seppic, Paris, France) for rabbit trials or Montanide PET GEL A (Seppic), a ready-to-disperse polymeric adjuvant designed to improve the safety and efficacy of vaccines for companion animals (Parker et al., 2009) for dog trials to a final protein concentration of 250 μg/ml (Merino et al., 2013; Contreras and de la Fuente, 2016, 2017).

### Vaccination Trials in Rabbits

Three two-year-old rabbits per group were assigned to *I. ricinus* or *D. reticulatus* trial injected subcutaneously (dorsum between shoulders) at days 0 (T1) and 14 (T2) with 0.2 ml (50 μg) doses of recombinant protein vaccine formulations or adjuvant/saline using a syringe with removable needle (0.45 × 13 mm). Two weeks after the last immunization (day 28; T3), rabbits in vaccinated and control groups were infested with 200 *I. ricinus* or *D. reticulatus* tick larvae of approximately 21 days after hatching placed on bags located on each rabbit’s shaved ear as described previously (Contreras and de la Fuente, 2016, 2017). Immunizations, tick larval infestations, collections and evaluations were done blinded and the key to the experimental groups was not disclosed until the end of the experiment. Engorged tick larvae were collected, counted and weighted as they dropped off between days 31 to 34. Fed larvae were incubated at 21°C, 80–82% humidity with 7 h dark and 17 h light photoperiod until molting. The tick larvae successfully molting to nymphal stage were collected and counted between days 51 to 65. The number of engorged larvae, weight/larvae, and percent of larvae molting to nymphs were evaluated. Data were analyzed statistically to compare results for each tick species between individuals fed on vaccinated and adjuvant/saline injected control rabbits by Student’s *t*-test with unequal variance (*P* = 0.05). Vaccine efficacy (E) was calculated as E (%) = 100 [1−(DL × DM_*N*_)], where DL is the reduction in the number of engorged larvae and DM_*N*_ is the reduction in the percent of larvae molting to nymphs in ticks fed on vaccinated rabbits when compared to the controls fed on adjuvant/saline injected rabbits (Contreras and de la Fuente, 2016, 2017). Only parameters with statistically significant differences were included in vaccine E calculation. Results were included in Supplementary Dataset 6.

### Vaccination Trials in Dogs

Three randomly mixed breed male and female dogs (age > 6 months; body weight > 2 Kg) per group were assigned to *I. ricinus* or *D. reticulatus* trial and injected subcutaneously (loose skin on the side of the chest) at days 0 (T1) and 21 (T2) with 0.2 ml (50 μg) doses of recombinant protein vaccine formulations or adjuvant/saline using a syringe with removable needle (0.45 × 13 mm). Two weeks after the last immunization (day 36; T3), dogs in vaccinated and control groups were infested with *I. ricinus* or *D. reticulatus* 25:30 (female:male ratio) adults of at least 10 days after molting and 100 nymphs of approximately 15 days after molting placed on feeding chambers (one chamber per tick stage) glued on each dog’s shaved flank. Immunizations, tick infestations, collections and evaluations were done blinded and the key to the experimental groups was not disclosed until the end of the experiment. Engorged tick females were collected, counted and weighted as they dropped off between days 41 and 48. Fed females were incubated at 21°C, 80–82% humidity with 24 h dark photoperiod for approximately 1 month until oviposition. Egg mass per tick was weighted and incubated for fertility determined by counting the number of larvae per tick. Engorged tick nymphs were collected and counted as they dropped off between days 40 to 43. Detached engorged nymphs were incubated at 21°C, 80–82% humidity with 8 h dark and 16 h light photoperiod until molting. The tick nymphs successfully molting to adults were collected and counted approximately 1 month after collection. The number of engorged nymphs and adult females, weight/female, oviposition (egg mass/tick), fertility (No. hatching larvae), and number of nymphs molting to adults were evaluated. Data were analyzed statistically to compare results for each tick species between individuals fed on vaccinated and adjuvant/saline injected control rabbits by Chi^2^-test (*P* = 0.05). Vaccine efficacy (E) was calculated as E (%) = 100 [1−(DN × DM_*A*_ × DT × DO × DF)], where DN is the reduction in the number of engorged nymphs, DM_*A*_ is the reduction in the number of nymphs molting to adults, DT is the reduction in the number of engorged female ticks, DO is the reduction in oviposition, and DF is the reduction in fertility in ticks fed on vaccinated rabbits when compared to the controls fed on adjuvant/saline injected dogs. Only parameters with statistically significant differences were included in vaccine E calculation. Results were included in Supplementary Dataset 6.

### Analysis of IgG Antibody Response by ELISA and Western Blot

In rabbits, blood samples were collected from each animal before each immunization (T1 and T2) and tick infestation (T3). In dogs, blood samples were collected from each animal before each immunization (T1 and T2), tick infestation (T3) and at day 75 (T4) to better evaluate the duration of the antibody-mediated immune response in the major vaccine target species. Blood samples were collected into sterile tubes and maintained at 4 °C until arrival at the laboratory. Serum was then separated by centrifugation and stored at −20°C. An indirect ELISA test was performed to detect IgG antibodies against recombinant antigens in serum samples from vaccinated and control animals collected at T1–T3 for rabbits or T1–T4 for dogs as described previously (Merino et al., 2013; Contreras and de la Fuente, 2016, 2017). High absorption capacity polystyrene microtiter plates were coated with 50 μl (0.02 μg/ml solution of purified recombinant proteins) per well in carbonate-bicarbonate buffer (Sigma-Aldrich). After an overnight incubation at 4°C, coated plates were blocked with 200 μl/well of blocking solution (5% skim milk in PBS). Serum samples or PBS as negative control were diluted (1:1000, 1:10,000, 1:100,000 v/v; optimal dilution, 1:10,000) in blocking solution and 50 μl/well were added into duplicate wells of the antigen-coated plates. After an overnight incubation at 4°C, the plates were washed three times with a washing solution (PBS containing 0.05% Tween 20). A goat anti-rabbit or anti-dog IgG-peroxidase conjugate (Sigma-Aldrich) was added (diluted 1:3000 v/v in blocking solution) and incubated at RT for 1 h. After three washes with washing solution, 200 μl/well of substrate solution (Fast OPD, Sigma-Aldrich) was added. Finally, the reaction was stopped with 50 μl/well of 3N H_2_SO_4_ and the optical density (OD) was measured in an ELISA plate reader at 450 nm. Antibody levels in vaccinated and control animals were expressed as the OD_450nm_ (OD_animal sera_−OD_PBS control_) and compared between vaccinated and control groups by ANOVA test (*P* = 0.05).

For Western blot analysis, 10 μg of selected purified recombinant proteins and *Rhipicephalus microplus* Subolesin (Merino et al., 2013) as negative control were separated by electrophoresis in an SDS-12% polyacrylamide gel (Life Science, Hercules, CA, United States) and transferred to a nitrocellulose membrane. The membrane was blocked with 5% BSA (Sigma-Aldrich) for 2 h at room temperature (RT) and washed three times with TBS (50 mM Tris-Cl, pH 7.5, 150 mM NaCl, 0.05% Tween 20). Pooled sera collected at T3 from vaccinated dogs were used as primary antibodies. Primary antibodies were used at a 1:300 dilution in TBS, and the membrane was incubated overnight at 4°C and washed three times with TBS. The membrane was then incubated with an anti-dog IgG-horseradish peroxidase (HRP) conjugate (Sigma-Aldrich) diluted 1:15000 in TBS with 2% BSA. The membrane was washed four times with TBS and finally developed with TMB (3,3′, 5,5′- tetramethylbenzidine) stabilized substrate for HRP (Promega, Madrid, Spain) according to the manufacturer recommendations.

### Determination of mRNA Levels by Real-Time RT-PCR

The expression of selected genes was characterized using total RNA extracted from three separate pools of adult tick salivary glands (25 females and 25 males each pool) and larvae (500 larvae each pool) using Tri Reagent (Sigma-Aldrich) according to manufacturer instructions. Real-time RT-PCR was performed on RNA samples using gene-specific oligonucleotide primers (Supplementary Table 1) and the iScript One-Step RT-PCR Kit with SYBR Green and the CFX96 Touch Real-Time PCR Detection System (Bio-Rad, Hercules, CA, United States). A dissociation curve was run at the end of the reaction to ensure that only one amplicon was formed and that the amplicons denatured consistently in the same temperature range for every sample. The mRNA levels were normalized against tick *ribosomal protein S4* (*rps4*) as described previously using the genNorm method (ddCT method as implemented by Bio-Rad iQ5 Standard Edition, Version 2.0) (Ayllón et al., 2013). Normalized *C*_t_ values were compared between salivary glands and larvae by Student’s *t*-test with unequal variance (*P* = 0.05).

### Protein Membrane Localization for *I. ricinus* and *D. reticulatus* Candidate Protective Antigens

To characterize the subcellular localization of proteins encoded by selected genes, the proteomes of IL and DL were obtained as described above but purifying membrane proteins prior to analysis. Approximately 200 *D. reticulatus* and *I. ricinus* larvae were pulverized in liquid nitrogen and homogenized with a glass homogenizer (20 strokes) in 4 ml buffer (0.25 M sucrose, 1 mM MgCl2, 10 mM Tris-HCl, pH 7.4) supplemented with complete mini protease inhibitor cocktail (Roche, Basel, Switzerland). Samples were sonicated for 1 min in an ultrasonic cooled bath followed by 10 sec of vortex. After 3 cycles of sonication-vortex, the homogenate was centrifuged at 200 × *g* for 5 min at RT to remove cellular debris. The supernatant was then centrifuged at 12000 × *g* for 30 min at 4°C and the pellet fraction enriched in crude plasma membranes was collected, resuspended in 100 μl Laemmli sample buffer and applied onto 1.2-cm wide wells on a 12% SDS-PAGE gel. The MS/MS raw files were searched against the Ixodida (40,849 entries in June 2013) Uniprot database and the protein database of candidate protective antigens (Tables 1, 2) using the SEQUEST algorithm (Proteome Discoverer 1.3, Thermo Scientific) with the following constraints: tryptic cleavage after Arginine and Lysine, up to two missed cleavage sites, and tolerances of 1 Da for precursor ions and 0.8 Da for MS/MS fragment ions and the searches were performed allowing optional Met oxidation and Cys carbamidomethylation. A FDR < 0.01 was considered as condition for successful peptide assignments. Results are shown in Tables 1, 2.

## Results and Discussion

### Algorithm for Selection of Genes Encoding for Candidate Tick Protective Antigens

According to Elvin and Kemp (1994) the conditions necessary and sufficient for an effective tick protective antigen include that (a) host antibodies should be able to gain access to the tick antigen, (b) sufficient antibodies must gain access to the target antigen, and (c) the formation of the antibody-antigen complex should disrupt the normal function of the tick protein. In our study, the hypothesis was that the genes encoding for functionally important secreted proteins that are highly represented in both L and SG would result in good candidate tick protective antigens. These antigens should fulfill the above conditions (a–c) as abundant functionally important secreted proteins would be highly exposed to host antibodies and their interaction should affect protein function, and thus tick feeding and/or development and pathogen infection and transmission.

To fulfill these criteria, an algorithm was developed using a vaccinomics approach to tick protective antigen discovery (de la Fuente and Merino, 2013: de la Fuente et al., 2016). The algorithm included the generation of transcriptomics and proteomics data in tick L and SG, and selection of gene transcripts and proteins highly represented in both samples and encoding for predicted functionally important secreted proteins. The initial dataset was obtained from *I. ricinus* because of the availability of the *I. scapularis* genome sequence for data mining (Gulia-Nuss et al., 2016). Genes selected in *I. ricinus* as encoding for tick candidate protective antigens (Table 1) were then used to find orthologous genes in *D. reticulatus* (Table 2). Tick IL and ISG were selected to cover both initial (larvae) and final (adult) developmental stages and tissues that are essential for pathogen acquisition and transmission, respectively (Ayllón et al., 2015; Chmelaø et al., 2016; Nuttall, 2018). *I. ricinus* and *D. reticulatus* acquire infection when larvae feed on infected hosts and after transtadial transmission, pathogen is secreted from salivary glands during feeding of nymph and adult ticks. Therefore, affecting these two developmental stages should reduce both tick infestations and pathogen infection/transmission.

### Selection of *I. ricinus* Candidate Protective Antigens

**FIGURE 1:**
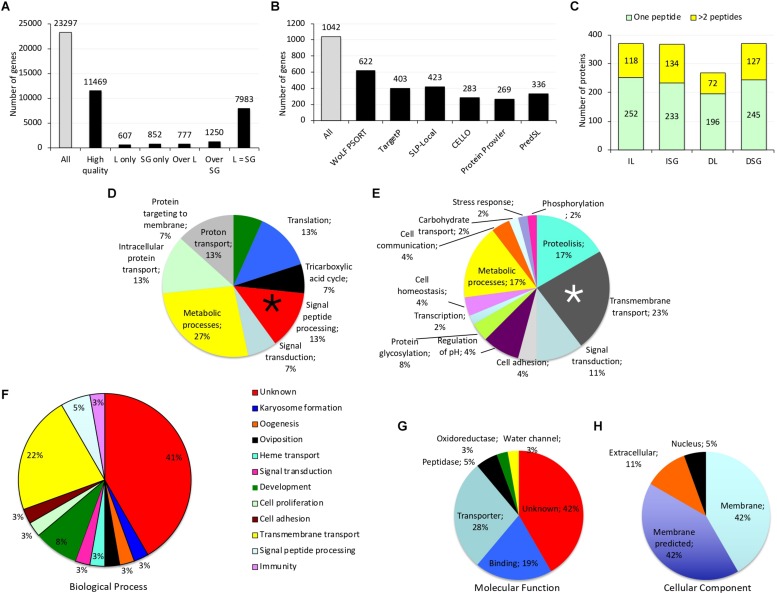
Tick transcriptomics and proteomics data. **(A)** Differential gene expression in *I. ricinus* larvae (L) and salivary gland (SG) samples. **(B)** Genes predicted by different algorithms to encode for secreted proteins in *I. ricinus* larvae (IL) and salivary glands (ISG) samples. **(C)** Proteins identified with XCorr > 2 in the Ixodidae database in IL, ISG, *D. reticulatus* larvae (DL) and salivary gland (DSG) samples. **(D,E)** Biological process (BP) GO for genes predicted as coding for secreted proteins that were either **(D)** among the top 1,000 expressed genes in both IL and ISG or **(E)** overexpressed in ISG. Genes coding for hypothetical proteins or with unknown function were excluded from the graph. The BPs selected for further analysis are marked with asterisks. **(F–H)** GO analysis for **(F)** biological process (BP), **(G)** molecular function (MF) and **(H)** cell component (CC) of genes selected as coding for tick protective antigens. Genes coding for nuclear DNA-binding proteins were removed from further analysis. Complete datasets are on Supplementary Datasets 1–4.

After RNAseq, the number of reads/sample corresponded to 27.7 and 27.2 millions for IL and ISG, respectively. Of them, 5,731,608 and 7,024,484 corresponded to pass filter high quality reads aligned to IL and ISG samples, respectively. Of the 23,297 identified gene transcripts, 49% were included in the analysis and not discarded due to not enough alignment, high complexity or shallowly sequenced (Figure 1A and Supplementary Dataset 1). Of them, 13% were expressed in IL or ISG only and 18% were overexpressed on either tissue, but most of the identified genes (70%) did not show differential expression between IL and ISG (Figure 1A and Supplementary Dataset 1).

**FIGURE 2:**
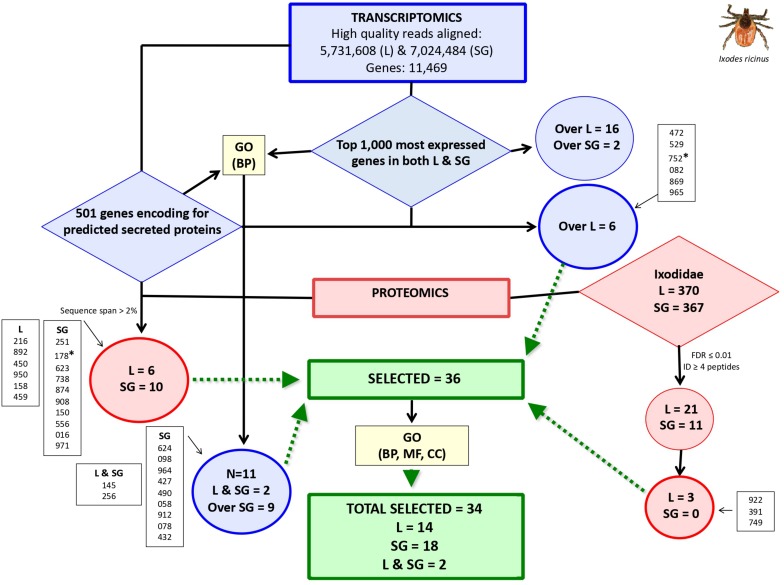
Selection of genes coding for candidate *I. ricinus* tick protective antigens. Trascriptomics data were obtained from *I. ricinus* larvae (L) and salivary gland (SG) samples followed by selection of top 1,000 most expressed genes in both samples and genes coding for secreted proteins. GO analysis for biological process (BP) was then used to select for functionally relevant genes. Proteomics data was used for mining databases of Ixodidae and predicted secreted proteins. Selected genes (*N* = 36; IDs. shown inside boxes) expressed or overexpressed (Over) in *I. ricinus* larvae (IL) and salivary glands (ISG) samples were finally subjected to GO analysis for BP, molecular function (MF) and cell component (CC) to remove two genes coding for nuclear DNA-binding proteins (marked with asterisks), resulting in a final set of 34 selected genes coding for candidate protective antigens. Complete datasets are on Supplementary Datasets 1–4.

Genes encoding for secreted proteins were predicted using different algorithms. Predictor’s performance was different with a total of 1,042 genes predicted as encoding for secreted proteins (Figure 1B). Protein Prowler performed rather poor (269 predicted proteins) and almost all of them (259 proteins) were identical to those predicted by TargetP. CELLO predicted a low number of secreted proteins. Moreover, it predicted as many as twelve different subcellular localizations, which made the results difficult to analyze. Therefore, the results from Protein Prowler and CELLO were discarded. Five hundred and one genes were selected as encoding for secreted proteins (Supplementary Dataset 2). These genes were predicted by at least two predictors. Of them, 208 (41%) were overexpressed in ISG and 97 (19%) were among the top 1,000 expressed genes in both IL and ISG with 6 of them overexpressed in IL (Supplementary Dataset 2). These 6 genes were selected as encoding for candidate tick protective antigens (Figure 2 and Table 1).

A total of 3,454 and 2,339 proteins were identified in the Ixodidae database in IL and ISG, respectively. Of them, after applying XCorr > 2, a total of 370 and 367 proteins were identified in IL and ISG, respectively (Figure 1C and Supplementary Dataset 3). An additional selection criterion using FDR ≤ 0.01 and protein identification with at least 4 peptides resulted in the selection of 21 and 11 proteins in IL and ISG, respectively (Figure 2). Finally, after filtering for redundant biological function, 3 genes were selected from IL as encoding for candidate tick protective antigens (Figure 2 and Table 1). The search against the database of predicted secreted proteins encoded by the selected top 1,000 expressed genes in both IL and ISG (Supplementary Dataset 4) using XCorr > 2 and sequence span > 2% resulted in the identification of 6 and 10 proteins in IL and ISG, respectively that were selected as candidate tick protective antigens (Figure 2 and Table 1).

The GO analysis of transcriptomics data resulted in different BP for genes predicted as encoding for secreted proteins that were either overexpressed in ISG or among the top 1,000 expressed genes in both IL and ISG (Figures 1D,E). In genes highly expressed in both IL and ISG, metabolic processes was the most represented (27%) BP (Figure 1D). In genes overexpressed in ISG, transmembrane transport (23%) and metabolic processes (17%) were the most represented BP (Figure 1E). Genes in the signal peptide processing BP (Figure 1D) and transmembrane transport (Figure 1E) were filtered for highly similar sequences and resulted in 11 genes (9 overexpressed in ISG and 2 highly expressed in both IL and ISG) that were selected as encoding for candidate tick protective antigens (Figure 2 and Table 1).

Finally, GO analysis for BP, MF and CC was conducted for the selected 36 genes encoding for candidate protective antigens (Figure 2 and Table 1). The results showed that approx. 40% of genes had unknown BP and MF (Figures 1F,G). As predicted, most of the genes (95%) encoded for membrane and secreted proteins, but two genes (ID Nos. 752 and 178) encoded for predicted nuclear DNA-binding proteins (Figure 1H). Although these two genes were initially predicted as encoding for secreted proteins, they were removed from further analysis due to the GO CC prediction (Figure 2 and Table 1). The most represented BP and MF corresponded to transmembrane transport (22%; Figure 1F) and transporter activity (28%; Figure 1G), respectively. Additionally, BP involved in development (8%), reproduction (oogenesis and oviposition; 6%), protein secretion (signal peptide processing; 3%) and immunity (3%) were also represented (Figure 1F).

**FIGURE 3:**
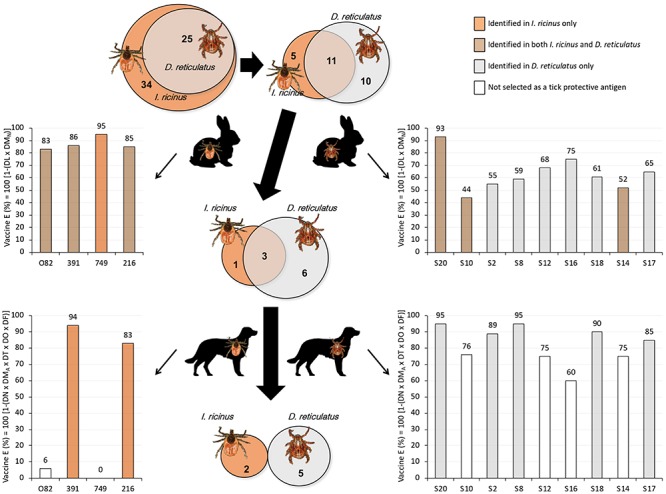
Pipeline for the identification of tick protective antigens. The Venn diagrams depict the set of genes selected for further analysis as coding for candidate tick protective antigens in *I. ricinus* and *D. reticulatus*. The graphs represent the vaccine E after vaccination trials in rabbits and dogs. Of the selected genes coding for *I. ricinus* (*N* = 34) and *D. reticulatus* (*N* = 25) tick protective antigens, only 16 and 21, respectively, were produced as recombinant proteins for initial vaccination screening in rabbits. After vaccination trial in rabbits, the criteria for the final selection of tick protective antigens for vaccination in dogs was based on vaccine *E* > 40%, resulting in 4 *I. ricinus* and 9 *D. reticulatus* antigens. The vaccination trial in dogs resulted in an overall vaccine *E* > 80% for 216 and 391 gene antigens in *I. ricinus*, and S2, S8, S17, S18, and S20 gene antigens in *D. reticulatus*. Full gene description and results are on Tables 1–3 and Supplementary Dataset 6.

The final set of genes coding for *I. ricinus* tick candidate protective antigens included 34 candidates (Figures 2, 3, Table 1, and Supplementary Dataset 4).

### Selection of *D. reticulatus* Candidate Protective Antigens

The number of reads/sample corresponded to 21.7 millions for DL and 21.8 millions for DSG. The 21,838,507 reads (∼2.2 Gb) in DSG were compared against the selected set of *I. ricinus* genes encoding for candidate protective antigens (Table 1), clustered and assembled to produce a set of contigs with homology to selected reference genes. These contigs were then analyzed for differential expression between DL and DSG samples and compared to results in *I. ricinus* (Table 2). The results showed that 16/27 (59%) of *D. reticulatus* genes had an expression pattern similar to *I. ricinus* (Table 2).

A total of 268 and 372 proteins were identified in the Ixodidae database in DL and DSG, respectively, and all remained after data filtering (Figure 1C and Supplementary Dataset 3). The additional selection criteria using FDR ≤ 0.01 and protein identification with at least 4 peptides did not produce new candidate tick protective antigens.

As previously described for *I. ricinus*, two genes (IDs S3 and S4) were removed from further analysis due to the GO CC prediction (Figure 2 and Tables 1, 2). Therefore, the final set of genes coding for *D. reticulatus* tick candidate protective antigens included 25 candidates (Figure 3, Table 2, and Supplementary Dataset 4).

### Selection of Tick Protective Antigens After Vaccination Trials in Rabbits and Dogs

Of the selected genes encoding for *I. ricinus* (*N* = 34) and *D. reticulatus* (*N* = 25) tick protective antigens, only 16 and 21 respectively were produced as recombinant proteins for initial vaccination screening in rabbits (Figure 3 and Tables 1, 2). Of them, 11 were identified in both tick species (Figure 3). After rabbit vaccination and infestation with *I. ricinus* or *D. reticulatus* tick larvae, the criteria for the final selection of tick protective antigens for vaccination trials in dogs was based on vaccine *E* > 40%. Selection criteria need to be applied in order to move forward in the vaccinomics pipeline of selecting candidate protective antigens, and *E* > 40% was set as the minimum vaccine E for tick larvae. The selection of tick protective antigens resulted in 4 *I. ricinus* and 9 *D. reticulatus* antigens (Figure 3, Table 3, and Supplementary Dataset 6). Of them, 3 were identified in both tick species (Figure 3). The results in dogs showed that vaccination with *I. ricinus* antigens affected different tick developmental stages (Supplementary Dataset 6) and resulted in an overall vaccine *E* > 80% for gene antigens 216 and 391 (Figure 3 and Table 3). Vaccination with *D. reticulatus* antigens mainly affected nymph feeding and molting and oviposition (Supplementary Dataset 6) with an overall vaccine E ranging from 75 to 95% (Figure 3 and Table 3). Finally, based on the criteria of vaccine *E* > 80%, which is considered a good vaccine E for selection of candidate protective antigens, the *I. ricinus* 216 and 391 and the *D. reticulatus* S2, S8, S17, S18, and S20 were identified as the most effective tick protective antigens (Figure 3 and Table 3).

**TABLE 3:** Results of the vaccination trials in dogs.

**Gene ID**	**DN (%)**	**DM_A_ (%)**	**DT (%)**	**DW_A_ (%)**	**DO (%)**	**DF (%)**	**E (%)**
***I. ricinus***							
082	6	0	0	0	0	0	6
216	20	5	33	5	0	67	83^*^
391	37	33	9	0	49	68	94^*^
749	0	0	0	0	0	0	0
***D. reticulatus***							
S2	40	41	0	22	69	0	89^*^
S8	64	60	0	0	63	0	95^*^
S10	10	9	0	1	71	0	76
S12	11	12	0	0	65	9	75
S14	0	0	0	0	75	0	75
S16	0	0	0	0	60	0	60
S17	41	50	11	0	43	0	85^*^
S18	55	60	0	0	43	4	90^*^
S20	55	63	0	6	67	14	95^*^

The number of engorged nymphs and adult females, weight/female, oviposition (egg mass/tick), fertility (No. hatching larvae), and number of nymphs molting to adults were evaluated. Vaccine efficacy (E) was calculated as E (%) = 100 [1−(DN × DM_*A*_ × DT × DO × DF)], where DN is the reduction in the number of engorged nymphs, DM_*A*_ is the reduction in the number of nymphs molting to adults, DT is the reduction in the number of engorged female ticks, DO is the reduction in oviposition, and DF is the reduction in fertility in ticks fed on vaccinated rabbits when compared to the controls fed on adjuvant/saline injected dogs. Only parameters with statistically significant differences (*P* < 0.05) were included in E calculation. All results are shown in Supplementary Dataset 6. ^*^Antigens with vaccine *E* > 80% were selected as the most effective tick protective antigens.

### Characterization of Identified Tick Protective Antigens

**FIGURE 4:**
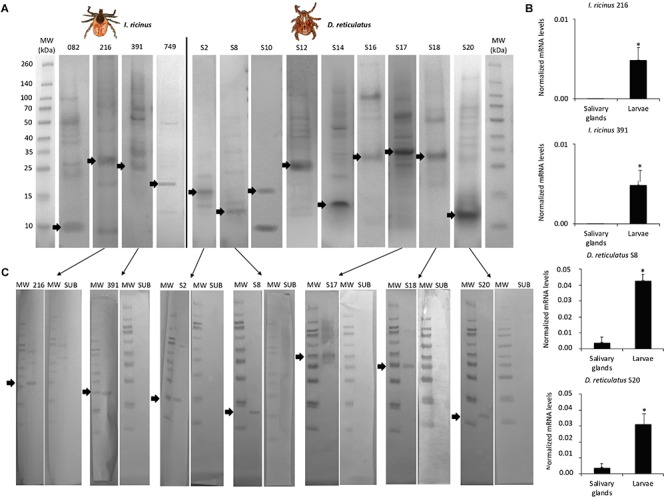
Characterization of candidate tick protective antigens. The 4 *I. ricinus* and 9 *D. reticulatus* candidate protective antigens were selected after vaccination trial in rabbits with vaccine *E* > 40%. **(A)** The histidine-tagged recombinant proteins were produced using the pET101/D-TOPO expression vector in *E. coli* BL21 Star (DE3) cells. After induction of gene expression with IPTG, collected cells were disrupted and the insoluble protein fraction containing the recombinant antigens as inclusion bodies were collected by centrifugation and purified by affinity to 16 polyhistidine. The eluted fraction containing the purified denatured proteins was dialyzed and concentrated by ultrafiltration to 0.5 mg/ml. Ten μg of purified recombinant antigens were separated by SDS-12% polyacrylamide gel electrophoresis and stained with Coomassie blue. Arrows indicate the position of recombinant antigens based on the predicted molecular weight. **(B)** The mRNA levels of selected genes coding for most effective vaccine antigens in dogs were determined by real-time RT-PCR in tick salivary glands and larvae (IDs refer to annotations in Tables 1–3). The mRNA levels were normalized against tick *rps4* and normalized *C*_*t*_ values (Ave+SD) were compared between salivary glands and larvae by Student’s *t*-test with unequal variance (^*^P < 0.05; *N* = 3 biological replicates). **(C)** Western blot analysis of selected recombinant proteins and Subolesin as negative control using pooled sera from vaccinated dogs collected at T3. Selected proteins were those corresponding to the most effective protective antigens with vaccine *E* > 80%. Arrows indicate the position of recombinant antigens. MW, molecular weight marker.

The candidate protective antigens were produced in *E. coli* and showed a molecular weight similar to that predicted by the amino acid sequence composition (Figure 4A and Supplementary Dataset 5). The mRNA levels of selected genes coding for the most effective protective antigens were determined by real-time RT-PCR and confirmed the results of the transcriptomics analysis (Figure 4B and Tables 1, 2).

**FIGURE 5:**
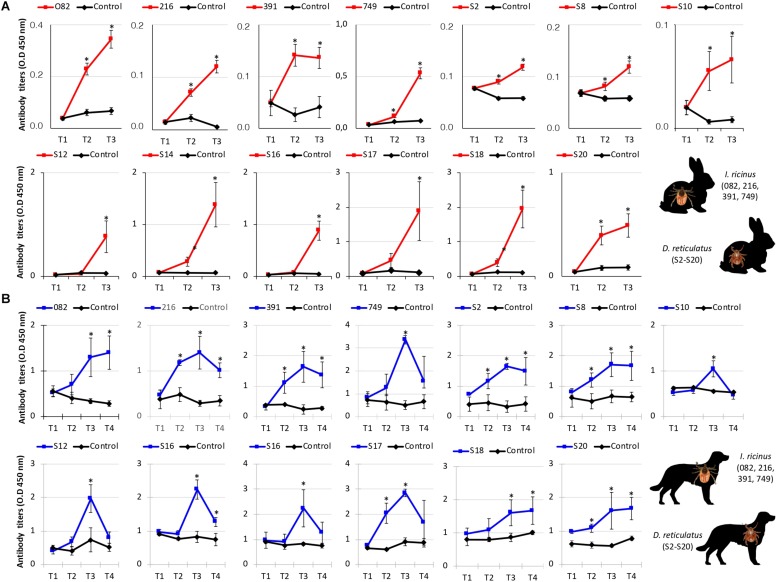
Immune IgG antibody response in vaccinated rabbits and dogs. An indirect ELISA test was performed to detect IgG antibodies against recombinant antigens in serum samples from vaccinated and control **(A)** rabbits and **(B)** dogs. Blood samples were collected from each animal before immunization (T1 and T2) and tick infestation (T3), and at day 75 (T4) in dogs. Serum samples or PBS as negative control were diluted at the optimal dilution of 1:10,000 (v/v). Antibody levels in vaccinated and control animals were expressed as the OD_450nm_ (OD_*animal sera*_–OD_*PBS control*_) and compared between vaccinated and control groups by ANOVA test (^*^P < 0.05). IDs refer to annotations in Tables 1–3.

The immunogenicity of candidate protective antigens (Table 3) was characterized by Western blot (Figure 4C) and indirect ELISA (Figures 5A,B) tests. The results supported the antigen-specific antibody response in vaccinated animals, and except for S2 and S8 antigens the background IgG levels were low in rabbits (Figure 5A). However, in dogs the background IgG levels before vaccination at T1 were higher than in rabbits (Figure 5B), suggesting host-specific differences in IgG affinity for tick antigens, and/or possible previous tick infestations at least in some of these dogs. Nevertheless, the IgG antibody response increased in response to vaccination in both rabbits and dogs, and for most antigens remained higher than in control animals until the end of the experiment (Figures 5A,B).

**FIGURE 6:**
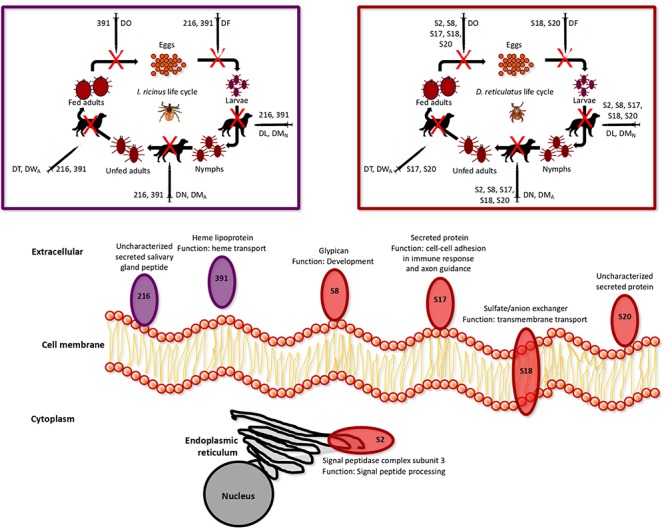
Summary representation of subcellular localization, function and vaccine efficacy of the selected protective antigens after vaccination trial in dogs. Complete GO annotations are in Supplementary Dataset 4, and the results of the vaccination trial in rabbits and dogs are in Table 3 and Supplementary Dataset 6. The *I. ricinus* antigens 216 and 391, and the *D. reticulatus* antigens S8, S17, S18 and S20 were identified in the membrane fraction derived from *D. reticulatus* and *I. ricinus* larvae. The effect of vaccination on tick life cycle was based on the parameters used to calculate vaccine E in rabbits and dogs, where DL is the reduction in the number of engorged larvae, DM_N_ is the reduction in the percent of larvae molting to nymphs, DN is the reduction in the number of engorged nymphs, DM_A_ is the reduction in the number of nymphs molting to adults, DT is the reduction in the number of engorged female ticks, DO is the reduction in oviposition, and DF is the reduction in fertility in ticks fed on vaccinated animals when compared to the controls fed on adjuvant/saline injected animals. Only parameters with statistically significant differences were included in vaccine E calculation.

Focusing on the selected tick protective antigens after vaccination trials in dogs (Table 3), the *I. ricinus* antigens 216 and 391, and the *D. reticulatus* antigens S8, S17, S18 and S20 were identified in the membrane fraction derived from *D. reticulatus* and *I. ricinus* larvae (Figure 6 and Tables 1, 2), thus confirming the prediction of the algorithms used for selection of membrane/secreted antigens. These antigens showed an effect on different tick developmental stages (Figure 6).

Among these proteins, *I. ricinus* 216 and *D. reticulatus* S20 are uncharacterized secreted proteins (Figure 6). However, heme lipoproteins such as *I. ricinus* 391 has been proposed as candidate vaccine protective antigens as being among the most abundant proteins involved in heme transport from hemolymph to tissues to complement the loss of the heme synthesis pathway in ticks (Braz et al., 1999; Maya-Monteiro et al., 2000; Lara et al., 2003, 2005; Hajdusek et al., 2009; Kluck et al., 2018). In agreement with this function, vaccination with 391 affected all the developmental stages during *I. ricinus* life cycle (Figure 6).

In *D. reticulatus*, a glypican-like protein S8 was identified (Figure 6). Glypicans have been used for cancer vaccines against hepatocellular carcinoma (Sun et al., 2016; Li et al., 2018), but not as protective antigens against ectoparasites or vector-borne pathogens. Glypicans are a group of cell-surface glycoproteins involved in the regulation of cellular morphology, growth, motility and differentiation (Li et al., 2018). The results of the vaccination trials suggested that these proteins might play a role in oviposition and development of immature tick stages (Figure 6).

The *D. reticulatus* secreted protein S17 was annotated as involved in homophilic cell adhesion via plasma membrane adhesion molecules and axon guidance (Figure 6). Proteins with cell-cell adhesion have a role in immune response and neuronal wiring, and are highly conserved across evolution (Samanta and Almo, 2015; Jin and Li, 2019). This protein family has been recently identified in ticks (Jin and Li, 2019), and our results suggested a role for this protein in all tick developmental stages except egg fertility (Figure 6).

Sulfate/anion exchanger molecules such as *D. reticulatus* S18 function as transmembrane transporters and are involved in different biological processes including regulation of blood pressure (Wall, 2016). Vaccination with this antigen affected larval and nymphal developmental stages, oviposition and fertility, suggesting a still uncharacterized function of this protein during these developmental processes (Figure 6).

The only protein with subcelllular localization in the endoplasmic reticulum (ER) was the *D. reticulatus* S2 antigen (Figure 6). This protein was annotated as a signal peptidase complex subunit 3, which is involved in protein targeting to ER through signal peptide processing. The disruption of the catalytic subunit of these conserved proteins leads to cell death in yeast (Bohni et al., 1988; Fang et al., 1997), *Plasmodium falciparum* (Tuteja et al., 2008), and *Leishmania manilensis major* (Taheri et al., 2010). Furthermore, knockdown by RNA interference of the gene coding for this enzyme in *Locusta migratoria* (*LmSPC1*) affected insect molting, feeding, reproduction and embryonic development (Zhang and Xia, 2014). Vaccination with this antigen reduced development of tick immature stages and oviposition, supporting the role of this protein in tick survival (Figure 6).

## Conclusion

The experimental approach used in this study resulted in the identification of tick protective antigens by using a vaccinomics approach in combination with vaccination trials. Based on the criteria of vaccine *E* > 80%, two *I. ricinus* and five *D. reticulatus* proteins were identified as the most effective protective antigens (Figure 6). Consequently, these antigens were proposed for commercial vaccine development for the control of tick infestations in companion animals, and other hosts for these tick species.

The putative function of selected protective antigens, which are involved in different biological processes, resulted in vaccines affecting multiple tick developmental stages (Figure 6). These results suggested that the combination of some of these antigens might be considered to increase vaccine efficacy through antigen synergy for the control of tick infestations and potentially affecting pathogen infection and transmission.

Finally, additional facts should be considered for vaccine development including that (a) a protective antigen is necessary but not sufficient for an effective vaccine as vaccine formulation, adjuvant and antigen composition including tick-derived and pathogen-derived antigens highly influence the final vaccine efficacy, (b) tick vaccines cannot be designed to prevent tick attachment and feeding because the immune response to vaccination (e.g., antibodies) needs to interact with target antigens in order to have an effect on different biological processes affecting life cycle of ticks and transmitted pathogens, (c) tick vaccines for wild animals are difficult to deliver, but appropriate vaccine formulations (i.e., virus-based) may address this problem, and (d) vaccines for companion animals may be acceptable if they increase animal welfare and health by reducing the duration of tick feeding and pathogen transmission.

## Data Availability

All datasets generated for this study are included in the manuscript and/or the Supplementary Files.

## Ethics Statement

White rabbits (*Oryctolagus cuniculus*) and Beagle dogs (*Canis lupus familiaris*) were used in the experiments. Animal experiments were conducted in strict accordance with the recommendations of the European Guide for the Care and Use of Laboratory Animals. Animals were housed and the experiments were conducted at LLC ACRO Vet Lab (Pylipovichi Village, Kyiv Region, Ukraine) with the approval and supervision of the Ukrainian Commission for Bioethics and Biosafety for animals under the studies “Tick vaccine experiment on rabbits” number 000369 and “Tick vaccine experiments on dogs” numbers 000576, 000577, and 000761.

## Author Contributions

MV and JdlF conceived the study and designed the experiments. MC and MV performed the experiments. All authors performed the data analysis, wrote the manuscript, and contributed to the final version of the manuscript and approved it for publication.

## Conflict of Interest Statement

The authors declare that the research was conducted in the absence of any commercial or financial relationships that could be construed as a potential conflict of interest.

## Footnotes

1 https://doi.org/10.5061/dryad.9js92

2 http://www.ncbi.nlm.nih.gov/nuccore/NZ_ABJB000000000

3 http://wolfpsort.org

4 http://www.cbs.dtu.dk/services/TargetP/

5 http://bioinf.scmb.uq.edu.au:8080/pprowler_webapp_1-2/

6 https://sunflower.kuicr.kyoto-u.ac.jp/s̃matsuda/slplocal.html

7 http://aias.biol.uoa.gr/PredSL/

8 http://cello.life.nctu.edu.tw

9 http://compgen.bio.unipd.it/bioinfo/software/

10 http://exon.niaid.nih.gov

11 http://amigo.geneontology.org/amigo

12 http://www.uniprot.org

13 http://www.ncbi.nlm.nih.gov

14 http://www.vectorbase.org/index.php
