# Supplementary material for: A Vaccinomics Approach for the Identification of Tick Protective Antigens for the Control of Ixodes ricinus and Dermacentor reticulatus Infestations in Companion Animals
Source: Front Physiol. 2019 Jul 26;10:977. doi: 10.3389/fphys.2019.00977 (PMC6681794; doi:10.3389/fphys.2019.00977)
Supplement: Supplementary file 5 [file Data_Sheet_5.pdf]

Supplementary Dataset 5. *I. ricinus* and *D. reticulatus* DNA and proteins sequences used for cloning and expression of candidate protective antigens (gene IDs refer to Tables 1 and 2).

I. ricinus

>472 (222 bp)
ATGTTGCTCCACCTCCATTGCTTTATTGCAAGAAAACTACATACAGCAACTCTCCTCATACAAT
TACAAGTGCCCATCACACTTTCGTTAGAAAAGAATCCAGGCAGCAAAAATGCGTGGGAGGACAC
GGCAGTTCCTCCTACGCTCCGGCAAAGGACCCACTTCGGTCCGGTCGGGAGAAACGGGGGGGAG
GGGAAGACGATAGCCACACAGATCCTAGAA

MLLHLHCFIARKLHTATLLIQLQVPITLSLEKNPGSKNAWEDTAVPPTLRQRTHFGPVGRNGGE
GKTIATQILE

>082 (195 bp)
ATGCCGAAACAAGGCGAAACCGCGAATGGCTCATTAAATCAGTTATGGTTCCTTAGATCGTTTC
TTCCTACTTGGATAACTGTGGCAATTCTAGAGCTAATACATGCAGTGAGCCCGAAGCCCCTCGG
GGCGACGGGTGCTTTTATTAGACCAAGATCAATCGGGCTTCGGTCCGTTTTGTGTGGTGACTCT
GGA

MPKQGETANGSLNQLWFLRSFLPTWITVAILELIHAVSPKPLGATGAFIRPRSIGLRSVLCGDS
G

>623 (465 bp)
ATGATAGGATGTATGCTGGTTGTCTTCATCGGGATCATCGTGAGCTTCATCACTGGTTACAAGA
AACCGTCTGATGTCAACCCGAGGACCGTCAATCCCCTCTATAACTTCCTCTGCAAGAGGTTTCT
TCCAAAGACACTGCACGACAAGTTTTTGGTCAAGTCCATAGAAGAGTACGACAAAGAGCAGATG
ATTACGCTCGGAAGATCCGGGTCTCCCGAGTTCACGCCGAATTCGAGCAACGGAAAAGTGAATC
CTGCTTTCGTGGCGAGCGACGACAAAGAGCTGGCCCTTGTGTCGTCGGCGAGGGACGCCACCAA
CGTCGAAGCGACGACGACGCCCGAATTAAAAGCGGTGCGAGCACTTGGCGATTCTCCGATCTCA
GACCGACGGAAAACGGCGTCCAACGGCGTCACGGGATCCCCAAACAAGGCCCTTTCAGAAGACG
AAGCTGCGACGCCGTTG

MIGCMLVVFIGIIVSFITGYKKPSDVNPRTVNPLYNFLCKRFLPKTLHDKFLVKSIEEYDKEQM
ITLGRSGSPEFTPNSSNGKVNPAFVASDDKELALVSSARDATNVEATTTPELKAVRALGDSPIS
DRRKTASNGVTGSPNKALSEDEAATPL

>908 (801 bp)
ATGGTGCCACCGACACTAGGAGCCGTGTTGCTACTTTGCGTCGTTGGAGCAGTGAATGCGGTCC
AGACCAACGAGGAGATTCCGGGTAAGGGTATCCTCCTGGACCCCCGATCCAAGTCGCTGGTGCT
CCAGTGCAACTCCACCGAAAACCCTGGCCTGGAAATTATATGGTTCAAGGACAACAAGAAGCTG
TCGGCCGACGAACCCAGGTACAGCCAGAACAGCGACACCAACACACTCGTCATCAATGACCCAA
CTTATGCGGACACCGGAAATTACACCTGCGCCGTGGTCGATGGAGGCGACAACGCCACCATTGT
GGTCCAGACCAACGTGACCATCGAAGTGTCCGAGATCTCGAAGAACCAAGTGGAAGGCGACCCC
CTGACCCTCTCCTGCAAGGCCTTCGGGGTTCCTGTGCCAGAAGTCACCTGGTTCAAGAATGACG
AGCCTCTCAACATCTCCGACCCCCGCATCTCCCTGGAGCCCCTGGACAACGTCACCAACGCCAA
GCTGGTGATCCAGAACCTCAACTTCGACGACCGCGCAGAGTACACCTGCTTCGCCACCAACGGC
ATCAGCAACGCCACCATGATGGTCCTGGTCCGGGTGAAGGACAAGCTTGCTGCCCTCTGGCCCT
TCCTCGGGATTTGCGTCGAAGTGGCAGTGCTCTGCGCCATCATCTTCATCTACGAGAAGAAGCG
CGTCAAGCCCGACTTCGAGGAGTCGGACACGGACCAGAACCCAGAGAACAAAAACTTGTCAGAC
CAGAAGGAAGGCCAGGACATCAGGCAGCGGAAG

MVPPTLGAVLLLCVVGAVNAVQTNEEIPGKGILLDPRSKSLVLQCNSTENPGLEIIWFKDNKKL
SADEPRYSQNSDTNTLVINDPTYADTGNYTCAVVDGGDNATIVVQTNVTIEVSEISKNQVEGDP

LTLSCKAFGVPVPEVTWFKNDEPLNISDPRISLEPLDNVTNAKLVIQNLNFDDRAEYTCFATNG
ISNATMMVLVRVKDKLAALWPFLGICVEVAVLCAIIFIYEKKRVKPDFEESDTDQNPENKNLSD
QKEGQDIRQRK

>016 (1233 bp)
ATGTTTATTTGCAGATTACACCATGCCCCCGTCTTCCTGTACACGTTACTTCCCCTCACCGGAG
CGGCAAGCGACCTCAAACACATCGACGATTCGCTCATGTTCAGCATCAACTGGCCTGGATCCAC
AGCAGACGACATAGGCAATGGGAGACTAGCGAGCGATCAGCCAGATCTAACTAAGCAGGAGGAT
CAGGAGTCGCTATGGGTTGTGTCTGCGGACAATGAACGATACCAGTGCATGCTTCCAAAGACAC
TGACCTCAAAAACGGATGCTGACCGCTCAACCCCATACACAGGACCGTCTCCATTGATGCTGCT
GAAGCCATTATTCACAAGAATGTTTTGTTCATATAAGTTGGAGCAGTACTGGACGTATGAACTG
TGTCATGGAAGGAGCATCAGGCAATACCACGAAGAAAACCAGCCCAGCAAGAATCGTCAATTTC
AGATCGTTCTGCAACAGTTCCACCTGGGGAGATACGACGCAGACAAAATGGAAAAAGACGAGGC
CGAGTACTTGCAGCAGCTCAAGTCAAGGCAAGAGGCTTCGAATAAGAAAGTGCGTCCCCCGACG
ATGCGGCTGGAGGGACTCGAGATGCCCTACTTCACGGTGAACATGACGGACGGCACCATGTGCG
ACATCAACAATGTGCGCCGCATGACGAGCGTCCTGTACGTGTGCAGCGAGGACTCCCGCAACGA
CATCCTCAGCCTCGAGGAGGTCTCCACGTGCGAGTACCAGGTGGTGGTACTCACGCCGTACCTG
TGCGCCCACCCAGACTACCGACTGGACTTTGCACCCGAGAACCACATCAGCTGCATCGCCAAGG
ACGGCGCTCCAACCAGGCCCAAGAGGATGGTGCTGATGGATCAGGAAGCCCACGGACTTCTGTA
TCCTGAGGAACAGAGTCCCAGGTCGCCGAGAGAGGACAGGCCGACCGTCGTCGACCCGGTGAGT
GTCAGCGGCAGTGGCGCCCCTCCGCACGACCCGAAGCTCCTCCAAGACTTTCTCAGCGGTGAAC
ACTGTCTCACCGGGGGAGTTGGCTGGTGGAAGTACGAGTTCTGCTACGGAAAAAGGGTCACCCA
ATTTCACGAGGAGAAAGACAAACCCCGCACCAGCATCCTGCTTGGAACGTGGGACAAGAAGAGT
CACGTGACGTGGATCGAGGAGCGCTCGGAGGAACGAACAAGGAAGGATCTAACAAGTCGTCGAC
CTCTTACTACTCGTGGG

MFICRLHHAPVFLYTLLPLTGAASDLKHIDDSLMFSINWPGSTADDIGNGRLASDQPDLTKQED
QESLWVVSADNERYQCMLPKTLTSKTDADRSTPYTGPSPLMLLKPLFTRMFCSYKLEQYWTYEL
CHGRSIRQYHEENQPSKNRQFQIVLQQFHLGRYDADKMEKDEAEYLQQLKSRQEASNKKVRPPT
MRLEGLEMPYFTVNMTDGTMCDINNVRRMTSVLYVCSEDSRNDILSLEEVSTCEYQVVVLTPYL
CAHPDYRLDFAPENHISCIAKDGAPTRPKRMVLMDQEAHGLLYPEEQSPRSPREDRPTVVDPVS
VSGSGAPPHDPKLLQDFLSGEHCLTGGVGWWKYEFCYGKRVTQFHEEKDKPRTSILLGTWDKKS
HVTWIEERSEERTRKDLTSRRPLTTRG

>216 (687 bp)
ATGAGGACCTTCGCCCTTTTCGCCGCGATTATCGCCGTCGCCGCCTACCAAGTGCACGGCCAGG
CTTGCCACCTTCGGGAGCTGGACCTCTGTGCCGCCTCCCTTCTGCTCTTTAACCAGAACCCATC
TGGTGTGGCCACCACCGACGCCGANNTGGACAAGCAGTGCGGTTTCCTGAAGGAGTCCCAGGAA
TGTTTCAAGAACTTCACTACTCGTTGTACCACTCCTCTGCAGAGGGAGCTCATCGGTTTTGTCT
CTGAGGGTTCCCAGGAACTCTTCAAGCAGTTCTGCTCCAAGGGCACTGAGATCAGGACCAACTA
CCTGAAGCACGCCCCTTGCCTCGGCCAGACCCTTCCCCAGCAGAAGCTTTGCCTTACCGACATC
CAGGCTGGTCTTGAGAAGATCGCCGTTGTGCCCTTCAACGACCGTGTCCCTGCCGCTTGCTGCA
TGTACTCCAGGTACCAGGCCTGCACCCGCAAGGCCATCACCGAAAAGTGCGGCGCTGAGGCTAT
TGAGTTCGGCGAGATCCTGGTGAAGATGGCCGCTTCCGACCTTCCCAACGTCGTGTGCAACTCC
TTCGACGCCAAGAACCCCAGGTGCAGTGCTCTGCTGCCACCCCCAGGCACCAAGCCCACCGGAA
AGTCCAACTCCGTCCTGTCCCGTCTTTTCTCCGCCTACCTGGGCAAC

MRTFALFAAIIAVAAYQVHGQACHLRELDLCAASLLLFNQNPSGVATTDAXXDKQCGFLKESQE
CFKNFTTRCTTPLQRELIGFVSEGSQELFKQFCSKGTEIRTNYLKHAPCLGQTLPQQKLCLTDI
QAGLEKIAVVPFNDRVPAACCMYSRYQACTRKAITEKCGAEAIEFGEILVKMAASDLPNVVCNS
FDAKNPRCSALLPPPGTKPTGKSNSVLSRLFSAYLGN

>158 (1245 bp)
ATGCGCTTCCTGAGCCTGCTGTCCGATGCCGAGCGCAACACGCAGCTGCTCTTCTCGAACGTGT
ACGCGGAGCGCATGGCGGCTGCGGCACGCGCCCCCGTGCGGAGCCTGTTCGCGGCCTTGGAGTC
CCACCTGCTGGGGACGCCGGGGCCCGCGGCCGAGGCCCAGCTGGGAGCCTTCTTCGATGAACTC

TTCCCCTTCGTCTACTTCCACACCGTCAACCCCAAGCTGAACGACTTCAGCGACGACTACAAGG
CCTGCCTGCGCTCGGCCCAGGGGCAGCTCAGGCCTTTCGGGGACGTTCCCACCAGGCTGCAGGG
CCCCCTGCTGGAGTCTCTGGGTGCAGCGCGGACGCTGCTGCGCGCCACCCGCGTGGCGCTGGAG
GTGCTGAACGCGTCCAGCGTGCCGGACTCCACGCCGACGCCCGGCTGTGGCCGCGCCCTGGCGC
AGCTCTTGGGCTGCCCCGCGTGCCTGGCCCCGCAGCACTCGCGCGGAGAGCTCCGACCCTGCTC
AGGCCTATGCCTCAACGCCCTGCGCGGCTGCCTGGCCGGCGTCGAGGACCTGGCGGCGCCCTGG
AGCGACCTGGTGTCGGCACTGCACCGTATGCTGCTGCGCATGGTGGGCTCCAACGACCTGGAGG
AGGTTCTGTCCGTCCTCGACTCCAAGGTGTCTGAGGCCGTCATGCATGCCATGGAGAACGGGCC
GGAGCTCTCCAAGAGGGTGAAGCTGGAGTGCGGGGACCCCAAGCGGCGACTGGGCAACGCCAGC
TCGGCGGAAGTGGCTGGACCGCGCAGAGTGACGGCGGCCCTGCGCAGCCCGAACACGTCCCTGC
GGACCCAGCAGCGCCAGTTCATGCAGCGGCTGAACGACTCTCGCACGCTCTTCTCCTCGCTCTC
CGAGACCTTGTGCTCGGGGCGCGGGATGGAGGCCACTCACGACACGCCCTGCTGGAACGGTGCT
GGCAGCGGGGAGTACACCAAGACTGTGGCGGGGGTGGGGACGTCAGCCCAGAAGTCCAATCCGG
AGGTGTCTTACGAGACGTCTTTGTGGGACTCCAGACTGGATGTCCTGGTGCACAAGCTCAATGA
CATGACCACTATCCTGGGAAACCGAATCTCCGTGATGCCAGAGTCGGACTCGTACACGATGGAA
GGCAGTGGCAGCGGGGCCTGGGGCAATGGGGAAACGTCGGACGACGAGGACTTTGACGGAGGTT
CGGGCAGCGGCATGGGTAAGTCTCAACTG

MRFLSLLSDAERNTQLLFSNVYAERMAAAARAPVRSLFAALESHLLGTPGPAAEAQLGAFFDEL
FPFVYFHTVNPKLNDFSDDYKACLRSAQGQLRPFGDVPTRLQGPLLESLGAARTLLRATRVALE
VLNASSVPDSTPTPGCGRALAQLLGCPACLAPQHSRGELRPCSGLCLNALRGCLAGVEDLAAPW
SDLVSALHRMLLRMVGSNDLEEVLSVLDSKVSEAVMHAMENGPELSKRVKLECGDPKRRLGNAS
SAEVAGPRRVTAALRSPNTSLRTQQRQFMQRLNDSRTLFSSLSETLCSGRGMEATHDTPCWNGA
GSGEYTKTVAGVGTSAQKSNPEVSYETSLWDSRLDVLVHKLNDMTTILGNRISVMPESDSYTME
GSGSGAWGNGETSDDEDFDGGSGSGMGKSQL

>459 (1275 bp)
ATGGCCAAGCTCCTCGCGGCCATTGTGGGATTCCTGGACAAGCAGAACTTGCTGTTTGTCGAGA
CTGCTGACATCTTGGCCATGATGGCGAGAGAGACACTGGTGCAGGCCAGGCTGCCCAGTTTCCA
CATACCATGTGCGGTTGAGGTCTTGACCCTAGGCACCTACTCCAGGCTTCCGACCTGCATCCGT
GTGTCTCTAACGCTGATGGGAGATGGACCGCACATTCCCTGGAGGCTGCTCGACATTGACATCC
TCGTGGAAGACAAAGACACCGGAGACTGCCGTGCCCTGGTACATTCACTACAGATCCAGTTTAT
TCACCAGCTGATACAGTCGCGCCTGGTCGACAACCCGAAGCCACTGCACGAGCTCTACAACTGC
CTGCGTATCCTTGCGGGCACCCCTCTTGCGTACTTGCAAGACTTTGCCGCGAGCGACTCGGAGA
TTTCGTCCGCGTCGATGAATATCGTCCAGGGCAAAATCTGGTTGTGCAGTACTGGAGATGACGA
GTTTATCTTGATGCGGCTCCGACACCTGCACTGCCAAGCGACTCTGATTGGAGGCTTGGGAGCT
GTGGTACTTCGTGACAGCTCGCGACTCTACCATGTTGTTGAGGGCTCTTCTCTTTTTGCCCGCA
GGGACCAGTTCAGCAAGGAGAAGCAGACGTACCAGCTGAACATCCAGGTGGATTCGGTGGATCC
CAGGAAGCCTCTCCGCGTGACCCACAACCCGGCGCTTCCGCACAAGGATGCTGTCTGGGCCGAC
CAGGCCATCAAGTCGGAGTTTCTGTCCGTGGAGAAGCTTCTCATCCAGACGATCCACATCCGGA
CCAAGCAGCGCCTGTCCGACCTTCGTGACAGACTCCGCAGCTCGGTCGTGGGACCCGCCGAATG
CCCCATTTTGGGTTCCCCAGCCATGCTGCAGGTTCCCCTGTTGCAGCCCTGCATGCAGTCGGAA
AACCTGCTCGTCACCGTGGACACCCACACCGGATACTTCCTGGCCTTTGTGCCCCAGTACGATC
CTCCCATGATTGGAGAGATCCAAGAGGCCCTGAACAAAGACGCGGCGAAGCTAGACACCCTCCT
CACAGACCTGAGGTTCTGGATGACGGTAAAGCGCTGTGAGAAGACGCTGCAGCACCTGCCCGTG
CTGACCAGTCCCAAGCTGCCCCTGGTGGTGCCCAGGGGTCATCCGGCCACCCGGCTGGGACCTC
ACACCCTCTATGTCAAGCTATGCAAGCACCACAACTGCTACGTGGTGAGCCAAGACGTC

MAKLLAAIVGFLDKQNLLFVETADILAMMARETLVQARLPSFHIPCAVEVLTLGTYSRLPTCIR
VSLTLMGDGPHIPWRLLDIDILVEDKDTGDCRALVHSLQIQFIHQLIQSRLVDNPKPLHELYNC
LRILAGTPLAYLQDFAASDSEISSASMNIVQGKIWLCSTGDDEFILMRLRHLHCQATLIGGLGA
VVLRDSSRLYHVVEGSSLFARRDQFSKEKQTYQLNIQVDSVDPRKPLRVTHNPALPHKDAVWAD
QAIKSEFLSVEKLLIQTIHIRTKQRLSDLRDRLRSSVVGPAECPILGSPAMLQVPLLQPCMQSE
NLLVTVDTHTGYFLAFVPQYDPPMIGEIQEALNKDAAKLDTLLTDLRFWMTVKRCEKTLQHLPV
LTSPKLPLVVPRGHPATRLGPHTLYVKLCKHHNCYVVSQDV

>922 (4932 bp)
ATGAGGGTGCTCGTAGCCCTCGCGTTTGTCGCAGCCGTGGCGGCCGCCGTTGAGGTGCCCCAAG
TGCTTCCAGAACTCCTCTTCGAGCCGAACCAGGAATACCTGTACAAGTACCGGACCGCGGTGTC
CCTGAGCCTCCCGCTCAAGGCCACGCACGCTACTGGAGAGGAAACCTACGGTCTGCTGAGCGTC
GTCGTCAAGGAAGCGTCCGGCACCGGCCGCAGTCTCGTCCTCCAGCTCCTCAACGTCACCTCGA
CGCTGTACGACAAGGAGGTCGAGGACCAGACTGAGCCAGTGCCCGGCGTCTACCACCAGCCCCT
GCCCGTGTTCGAGTCCTACCAGACCGGACCCGTGGTGCTGAAGCTTGTCGACCACAGTGTGGAG
AGCCTGGAGGTGCCCGTGGGCGTGCCTGAAGAGGTGGTGAACCTGTACCGTGGTCTGGCCTCCG
TGCTGACCCTGAGCAACCCCAGCTACAAGAAGGTGCCCTTCACCAAGGACGTGCCCCTGACCCT
CAAGGACGACGTCGTGGTCTACAAGGTCTACGAGGACGATCTGGTCGGTACTTGCGAGACCGTG
TACAACGTGCTGTCCAGCCCGCACGACGAGTACGTCCTGAACTTCACCAAGACCAAGAACTACC
ACAAGTGCGTGGGCAAGACCACCGTCTTCCAGCACGTCGACTACGAGCACAGCGGATGCCCCCA
CGCTTGCCTGAAGCACCAGCCCAAGGCCCTCAGTGAAACTCTGGAGCCTGAGCTTTCGGACTAC
GTCGACCCTTACGGAGGCGGCTGCCCCACCGAGACCCACCTGAAGAACGACCTTGCCGAGTCCT
TCCTGACCGTTCATTACAACGTCAGCCTTCACCAGGAGGTCGGCGTTCTTGAAGAAGTCAAGGC
TATCGACAAGAAGGTCCTCACCTCCGGAAAGCAACAGCTGGTTTCTACGTCCGTGCTGCACCTT
GAGCTGCTCCTCAAGACCACTCCGTTCACCGCCGTGGGTCCTCTGGAGGACGTCAAGACCTACA
CCAACCTGTCGTACGTTTACCCCAAGCAGCACTACTCCTGGCATGGACAGCTCTACGAGCTCGA
GCACCTGAGTCTGTACGGGCCCGTGGATACTGTGGAGGCCAGGACAGCCGTCCGTGGTCTTCTA
GACCAGCTCGCTGGTCTTTTGGTTCTCGACGACCTTGAGGTCAAGGATGACTACGCCGACCTCG
TGTCGCAGCTGCTGACCGCAGTCAATGTCCTCAAGGAGTACGACCTCGAGCTCCTGCTGCAGAC
GGTCGTTCCTCTGGAAAACGTCAAGGTTGTCAGCGAGAAGGAGTACATCGAGAGGAAGCTGCTG
CTTGACGTGCTCAGCCTTGCTGGTACCGACGCCGCCGCCAAGACGGTCCTCAGGCTCCTTCTTG
AGCAAAAGCTGACCCTCGTGGAAGCCGTCCACGTCCTCACTTCCCTCCAGACTTCCCTCGTGAA
GCCCAGCACCGAAGTCCTCGACTTGCTCCTGGACTTGGCCACAAGCGGAGTTCTCGAAAAGGAC
CGTCTCCTCTACTCGACGGCGTACCTCACACTGGCGAAGGTCGTGAGCAAGCACTGCCACCTGT
ACGACACCACGAGCCACGTCCCCTACGGCAAAATGCTGAAGATGAATGAGGTTGACGCGATCAA
GAAGAGACCGCCCCACCTGCCCACGTACCGGGCGATGAAGACGTACCGCCCCCGCATCTCCGGC
CGCCAGTACCAGGAGACTGAGACCGAGGAGCCCCAGTACACCGGCGTCCCCGTGACGTGCACCA
GCCAGGACTACCTCAAGTATGTCCAGGCTCTTGTCCAGAAGCTGAACGAGGCCAAGGAGTTCCA
CCAGGTCACCGTCCTGGTTCACGCCCTGACCCAGCTGCAGCACCCTGAGGCTCTCAAGGCCCTC
GTCCCCGTTGTCCTCGGCAAGCACCACCTCTGCCAGGCCACCCTTCCGGAGGAGGAACAGTCTG
AGTCCTGCCAGTACCTTCGTCTTGTCACACTGTACGCCCTGCGTCACAGTGTCAAGCACCATGC
TGCCGAACTCCAGCCCCTTGCCCAGACCGTCTACTTCAACACCGACGAGGACTACGAACTTCGT
AACGCGGCCCTGGTTCTCCTGATGGCCTCTCACCCACCCGAGCCGGTGCTGGCTCGCGTCGTCC
TCACCCTTCAAAAGGAGTTGAACCTGCAGGTGGCCTCGTTCACCTACTCTACGCTGTTCGCCTA
CGCCAACGCCACCGTTCCGGTCTCGAGACCCTCGGCAAGTACCTGCTCCGTCACGAAGGCCCAG
TCACCGTCTTCGCCGAAGCCTTCCAGAGGGTCCGTCGCCAGATCTTCGACAAGTACGAGACGTA
CGAGCCACTCAGCCAACTCCTCGACGTCCTCGAGAAGCAGCGTGTACACTTCGGCTGTCTCTTC
CGTGGTCACGGTGACTCCCTTCACCAAGGTCGTCGCCGGTGTGCGCACCCTGGCAAAGACCAGG
CTCACCCTGCCTTACGACTTCGCCTTCCAGTACTCGTCAGTGAACCACACCGTCGCCCTCACCG
TGCGCCCCCGCTACACCAAGTTCTTCACCCACCATACCAAGTCGGTGACGTATACCACGCCTGC
CGTGCTCTACACGCCGCCCCAGCGCGCTGTCATGGCCACCATGAGCGTCCTGTACAGTCAGGCC
AAGCCTTTTGAGTTCAAGAAGACCTTCGGACACGAAGCCCTCGGTCTTGGACTCCACGTCCAGG
GACTGTCCAGCCACCCCGACCTCCACGTGCCGTTCTCCTTCCAGCGCTACACCAAGGAGAAGGG
ACTCCTCGGTGCCTTCGTCGACGTCCTCACCAACCCGTGGCACGTGGCTAGGAAGTGGGAAGCT
CGTGTTGTCCGCAGCACCGTTCATCCCGTGGATGAGTACAAACTGACCTTCCGCTGGAAGACCA
CACTGAACCAGACCGACCGCACCGACTTTGTCGAGGAAGTGCTGCCGGCCCCGTACCAGTCCAC
CAAGTACCCTACCACAAGGTACGGCTACCCCGAGGACCTCGAGACGCCCATCTACGAGGAGCAC
TACAAGCGTGCCGTGGGAGAGCTCCCACCGTACTTCGACTTCTACCAAGTTCTCCAGGAGGTCG
TGGCCGCCACCGAGGTGACCAGCACGCCCCTGGCCCACTACGTGACGCCCTTCTTGAACGACGT
GCAAAACACCACCTACGGTTACTTCGTCGAGTTTGTGCTCGGTGCCCTCGGACCCCTTGAGACC
AAGGTGCTGACTGGACACGCTCTGCTCGGACATACCTTCGACAAGGCCCTCAAGCTTTCCCAGC
TCTACGTCAACAAGGTCAACAGCCCATACGAGGTGAAGGTGCACTCTGCCCTCCTCAAGACCGC
CGTCCCAAGCCCCTTCAAGTCCTACGCCGTCGACAAGAAGGAACAATCTCTCTACTTCACTTCC
GTTCTCGACGTCCTGACCGAAGAGAGCGACACGCAGAAGTATACTCTTCAGGTGAAGGTGCACT

CTGCCCTCCTCAAGACCGCCGTCCCAAGCCCCTTCAAGTCCTACGCCGTCGACAAGAAGGAACA
GTCCCTCTACTTCACTTCCGTTCTCGACGTCCTGACCGAAGAGAGCGACACTCAGAAGTATACT
CTTCAGGTTCCCGGACTTCTCAAGAACGTGACTCACAGCCTTCTCCAGTACGCCAAGGTCTTGT
TCTACAACAAGCTCGTCACCGACTACGACGTCGAGACGAGGTCGACCTTCACGCATCTCGAAGG
CGAACTTGTGTTCGAGGACCACCCGACAGGAAAGACCACCGCCAACCTGACGCTCTACACGCCG
CTCGAAGAGAAGCTTCAGTTCGTGCGCCTCAACTGGTTTAAGTACCTGAAGCCCACGGTCGCCA
TCGGCTTCCTGGACACAGTCGCCGGAGTCCTGAGGAGGAGCTACCCTACCCCCACCTGTCTGGT
GTCGCCTACCTACCTCAAGACCTTCGACAACGTCACCGTGCTCCTGAAGCCTTATTTGAGGGAC
GACACATACGTTGTTGCCCGTCACGTCCTCGACGAGCCAGACTTCTACGTTCTGACCGAGTTCC
AGGGCGAAACTCAGCTGGTGAAGCTGATCCTGCGGAACCAGACCCTTCTGGAGCTGACTCCTCC
TAGAGACGGCAAGACCTTCGAGGTCCTGGTGAACGGCACCAGCCTCTACGTGGAGCCGCTCAAG
TCGCACGTACTCCAGTACACGCAGAACTACACGTCTCAGGTCTTCCTGTACGTCACCGAGCACC
AAGAGGTCGGCCCGACCCTCTGGGTTACGGTCCGCGATCTTGGCCTCAAGTTCGCCTACGATGG
AAGCAAACTGATATTCAAGGTCCTCAGCCCCAAGTACAAGGGACGCGTCCTGGGTCTTTGCGGA
GACTTGGACGGAGAGTACGTGGACGAACTCGTCACCCCCGAGCTCTGCGTCCTCACTGAGGAGG
AAGACTTTGTGCAGACCTACAGCCTCAAGGGCCTCGAGTCCACGGTCGGTCTCTACAAGTGCCC
CCTCGGTGTCACCCTCCGCGGTGTTGGCTATCCTTCCTACCCCCGCATCCAGAAGACCTTCGCC
AGGATCAACCCGATCGAAGTTGAGAAGGAAATCGAGATCCCCAAGCTGATCGGCACCCCCGAGT
GCGTCACCGAGCGTCGTAAGACCATCTACAAGGACGGAAAGGTGTGCATCAGCACCAAGAAGGT
GACGGCCTGCCAGCGCCGCTGCAAGCCCGTGGCTACCGAGAAGGTGACCCTCGAGTTCGTCTGC
CTGAACGAGAAGCACCCGGTGGCCAAGAGGCTGCTCAAGGACATCCAGGGCAAGCGCAACGTTC
GTCTGCCCACGGACATCCCTACATTCAACGAGGACGTCGAAATCCCCACTGACTGCGTGCCAGA
GGTG

MRVLVALAFVAAVAAAVEVPQVLPELLFEPNQEYLYKYRTAVSLSLPLKATHATGEETYGLLSV
VVKEASGTGRSLVLQLLNVTSTLYDKEVEDQTEPVPGVYHQPLPVFESYQTGPVVLKLVDHSVE
SLEVPVGVPEEVVNLYRGLASVLTLSNPSYKKVPFTKDVPLTLKDDVVVYKVYEDDLVGTCETV
YNVLSSPHDEYVLNFTKTKNYHKCVGKTTVFQHVDYEHSGCPHACLKHQPKALSETLEPELSDY
VDPYGGGCPTETHLKNDLAESFLTVHYNVSLHQEVGVLEEVKAIDKKVLTSGKQQLVSTSVLHL
ELLLKTTPFTAVGPLEDVKTYTNLSYVYPKQHYSWHGQLYELEHLSLYGPVDTVEARTAVRGLL
DQLAGLLVLDDLEVKDDYADLVSQLLTAVNVLKEYDLELLLQTVVPLENVKVVSEKEYIERKLL
LDVLSLAGTDAAAKTVLRLLLEQKLTLVEAVHVLTSLQTSLVKPSTEVLDLLLDLATSGVLEKD
RLLYSTAYLTLAKVVSKHCHLYDTTSHVPYGKMLKMNEVDAIKKRPPHLPTYRAMKTYRPRISG
RQYQETETEEPQYTGVPVTCTSQDYLKYVQALVQKLNEAKEFHQVTVLVHALTQLQHPEALKAL
VPVVLGKHHLCQATLPEEEQSESCQYLRLVTLYALRHSVKHHAAELQPLAQTVYFNTDEDYELR
NAALVLLMASHPPEPVLARVVLTLQKELNLQVASFTYSTLFAYANATVPVSRPSASTCSVTKAQ
SPSSPKPSRGSVARSSTSTRRTSHSANSSTSSRSSVYTSAVSSVVTVTPFTKVVAGVRTLAKTR
LTLPYDFAFQYSSVNHTVALTVRPRYTKFFTHHTKSVTYTTPAVLYTPPQRAVMATMSVLYSQA
KPFEFKKTFGHEALGLGLHVQGLSSHPDLHVPFSFQRYTKEKGLLGAFVDVLTNPWHVARKWEA
RVVRSTVHPVDEYKLTFRWKTTLNQTDRTDFVEEVLPAPYQSTKYPTTRYGYPEDLETPIYEEH
YKRAVGELPPYFDFYQVLQEVVAATEVTSTPLAHYVTPFLNDVQNTTYGYFVEFVLGALGPLET
KVLTGHALLGHTFDKALKLSQLYVNKVNSPYEVKVHSALLKTAVPSPFKSYAVDKKEQSLYFTS
VLDVLTEESDTQKYTLQVKVHSALLKTAVPSPFKSYAVDKKEQSLYFTSVLDVLTEESDTQKYT
LQVPGLLKNVTHSLLQYAKVLFYNKLVTDYDVETRSTFTHLEGELVFEDHPTGKTTANLTLYTP
LEEKLQFVRLNWFKYLKPTVAIGFLDTVAGVLRRSYPTPTCLVSPTYLKTFDNVTVLLKPYLRD
DTYVVARHVLDEPDFYVLTEFQGETQLVKLILRNQTLLELTPPRDGKTFEVLVNGTSLYVEPLK
SHVLQYTQNYTSQVFLYVTEHQEVGPTLWVTVRDLGLKFAYDGSKLIFKVLSPKYKGRVLGLCG
DLDGEYVDELVTPELCVLTEEEDFVQTYSLKGLESTVGLYKCPLGVTLRGVGYPSYPRIQKTFA
RINPIEVEKEIEIPKLIGTPECVTERRKTIYKDGKVCISTKKVTACQRRCKPVATEKVTLEFVC
LNEKHPVAKRLLKDIQGKRNVRLPTDIPTFNEDVEIPTDCVPEV

>391 (582 bp)
ATGGTTAACTATATATCCTACTACAAACAAAAGCATTGCGACCTGCAAGGCAAGAGTGTCCGGA
CCTTCGACAACGTCGTCGTGAACCTTCCCGAGACTGACTGCTTCAAGGTGGTCGCCAAGGACTG
CTCTCCCAACAAGAAGTTCACGATCCTGGCACGAGCTACCGGAAACGCCGCACTCCCCAAGGCG
TTGAAGGCCTTCATACAGTCCACCAAGATCGAGCTCCTGCCAGTTTCCGCCGATTCCGGACTGG

TGCTCCGTGTTGACGGCAACAGGGTTCTACTGACGCAGGGAGTACCTTACAGCCACACCGCCCA
CGACGTGGAGCTATTCACGGTCACGCAGCACAACAAGTACTTCGAAGTGATGTCGCAGCCTTAC
GGGGTTTACATGGGCTTCGACGGAAACGCCCTCTTTGTGCAGACCGCCAACTTCTACCGCGGTA
AGCTGTGCGGCCTTTGCGGCGACTACAACTACGACCGGCAGCACGAGCTCGTGGGCCCCAACCT
CCACCACTTCAACGACACCCTGGAGTTCGCCAAGAGCTACGTCGTTCCCGCGTCTGACTGCACC
GCTCCC

MVNYISYYKQKHCDLQGKSVRTFDNVVVNLPETDCFKVVAKDCSPNKKFTILARATGNAALPKA
LKAFIQSTKIELLPVSADSGLVLRVDGNRVLLTQGVPYSHTAHDVELFTVTQHNKYFEVMSQPY
GVYMGFDGNALFVQTANFYRGKLCGLCGDYNYDRQHELVGPNLHHFNDTLEFAKSYVVPASDCT
AP

>749 (537 bp)
ATGATCGCCCAGGCTCCCCAGGCTAACAAGTTCGGCTACAACATCGTCGACAACTACGGCAACC
ACCAGAGCCGTCACGAAGTCTCCGATGCCCACAACCGTCGTGTCGGATCTTACAGCTTCACCGA
CGCTCATGGCCGTGCCCGTAAGGTCAGCTACGTCGCCGACGGACATGGTTTCCGCGCTGTCGTC
CACACCAACGAGCCCGGAACCGCCGCTTCCCGCCCCGCTGCCGCCGCCTACAACACCCCCGTCG
TCCACAAGGCCCCCCAGGCTAACAAGTTCGGCTACAACATCGTCGACAACTACGGCAACCACCA
GAGCCGTCACGAAGTCTCCGACGCCCACAACCGTCGTGTTGGATCCTACAGCTTCAGGGATGCT
CACGGCCGTGCCCGTCAGGTCAACTACGTCGCCGACGGACATGGCTTCCGCGCCGTCGTCCACA
CCAACGAGCCCGGAACCGCCGCTTCCCGCCCCGCTGCCGCCGCCTACAACACCCCCGTCGTCCA
CAAGATTGGAGTGACGTTCTTGAAG

MIAQAPQANKFGYNIVDNYGNHQSRHEVSDAHNRRVGSYSFTDAHGRARKVSYVADGHGFRAVV
HTNEPGTAASRPAAAAYNTPVVHKAPQANKFGYNIVDNYGNHQSRHEVSDAHNRRVGSYSFRDA
HGRARQVNYVADGHGFRAVVHTNEPGTAASRPAAAAYNTPVVHKIGVTFLK

>490 (882 bp)
ATGTTGGACGGATTCAAGATCAAGAATGCTGTTATTCGAGAATCTCTCGCCGAAATGCTGGGAA
CATTCGTTCTTGTGCTTTTCGGCAACACCGTTCTGGCGATTCTGACCTTCGAGAAAGCCAGTAC
CGACGGCCTGGCAGCCTGCTTCTGGGGATGGGGGCTGGCCCTCACCCTCGGAGTCCTGGTCGCC
GGTGGAGCATCCGGGGCTCATCTGAACCCGGCCATCACGGTTGCTGTGACGACGATAGGCAAGT
TTCCCTGGAGGAAAATTGTCCCCTACGTGATCGCCCAGTACATCGGAGCCTTCATCGCTTCCGT
CGTCCTCTTCATCACCTATCGAGGCGCCTTGGATAATTTTGATGGCGGGAACCGCATCGTCACC
GGCGTCAACGGCACCGCTGGCATCTTCGCATCCTATCCCAAGGAATTCCTCAGCACCGGAAACG
GGCTCGTCGATCAGATCGTGGGCACGGCGCTGCTTATGCTCTGCATCCTGGCCATCACGGACGC
GCGCAACATGGCGGTGCCCCAAGGTGTCCAGCCGCTGTTCATCGGCTTCGCGCTGGCCGCCATC
ATCCTCAGCTTCGGCTACAACTGCGGGGTTCCCCTGAACCCGGCTAGGGACCTCGCACCTCGAG
TCTTCACCGCCATGGCCGGATGGGGCGGCGAGGTCTTCAGCTTCCGGGACTACAACTGGTTCTG
GGTGCCCATTCTGGGACCCCACATCGGCGCCATCCTCGGTGCCTGGATCTACACCCTCGCCGTG
GAGCTGCACTGGCCCGGTGCCACGTACGAGATGGACGGCGGCAACCCCGTGTCCACCAAGGACG
TCGAGAACAGCGTCCAGATGCGAGGCATCAAGCTGGGCGACTCCAAGAAC

MLDGFKIKNAVIRESLAEMLGTFVLVLFGNTVLAILTFEKASTDGLAACFWGWGLALTLGVLVA
GGASGAHLNPAITVAVTTIGKFPWRKIVPYVIAQYIGAFIASVVLFITYRGALDNFDGGNRIVT
GVNGTAGIFASYPKEFLSTGNGLVDQIVGTALLMLCILAITDARNMAVPQGVQPLFIGFALAAI
ILSFGYNCGVPLNPARDLAPRVFTAMAGWGGEVFSFRDYNWFWVPILGPHIGAILGAWIYTLAV
ELHWPGATYEMDGGNPVSTKDVENSVQMRGIKLGDSKN

>058 (1546 bp)
ATGAGGGTGCCCATTGTGAAATGGCTGCCCAAGTACTCCCTGCTGGACCTCCACGGGGACTTTG
TGGCCGGCATGACTGTGGCCCTCACCGTCATCCCCCAGGGACTGGCACTGGCGGACCTCGCAAA
GCTCCCCATAGAGTACGGCCTGTACACAGCATTCATGGGTGGCTTCATGTACGCCATCTTCGGG
AGCTGTAAGGACCTGACGATAGGACCGACGGCCATCATGTCCATCATGACTGCGGAGTACGTGA
AGCATGGGGGACCAACGTACGCTGTCATTCTAACCTTCCTCTCGGGCATCATCCAGATACTAAT
GGGAGTTCTCAACTTAGGTTTCATTGTTGAATTCATATCCGGTCCGGTGATCAGCGGCTTCACC

TCTGCGGCAGCCATCACCATCGCCAGCACGCAGCTGGAGGTGAGCATTTTTTCTCTAGAAAACT
CTGATTTCGTGCCGCCGAGCATTTCTGATTTTTCTTTTATTTTGATGTGCAGGTTGGGTGACTC
TCTGCTTGGTGTGTCTTCTGTGATCTTGCTTTTGGTAGTGAGGCACTTCAAGGATTGCAAGTTC
AGTCAAGACTCGAGACTACCTCCAAAGGTCCGCAAGGTCATCGAGACTGCCTGGTGGACGATAG
CCACGGCACGCAATGCGATAGTGGTCTTGGCGTGCGCCATTCTGGCGTCGTGCCTCCTCAACAT
TGGCATGGAGCCCTTCGACCTCACCAAGGAAGTCCAGGGAGGACTTCCCTCCTTTCGGGTGCCT
GATTTCAGCGCCAATTTCAACGGCACCAACTCCACAACCATTCACAAGGACTTCTTCGACATTG
TCCAGGAGCTGGGTTCTGGCATTCCTATCATTGCTTTGCTGTCGATCTTGGAGTCTGTTGCAAT
TGCAAAAGCATTTGCCAAGGGCAAGACACTAGACTCCACGCAGGAAATGATGGCAATAGGCATA
TGCAACCTGATGGGGTCCTTCGTCAGCGCATACCCCGGAACTGGAAGTTTCTCAAGGACAGCCA
TTAACAACAACAGCGGCGTTCGAACACCAATGGGAGGAGTTTTCACAGGTACCATTGTGATCAT
GGCCCTGGTCTTCATGGCACCCTACTTCAAGTTCATCCCCAAGGCCTCCTTGGCGGCCATCATC
ATCACCGCCGTCATCTTCATGATACACTACCAGGACGTCCCCGGCATGTGGCGCACAAACAAGA
TCGACCTGTTCCCATTCACCGCCACTTTCCTCGTGTCCTTTGTGCTTGGATTGGAGTACGGCAT
CATCGCCGGTGTTGTCATCTCCCTGGCTCTGCTGATGTACGAACACGCCCGACCACGCATCAGG
ATCTCTCGCAAGACTACCCTCTCTGGTGTGCCGTACCTGCTGGTGTCCCCTGACCGCAGCGTGC
TGTACCCGTCCTCGATGTACACAAGCTCCAAGATCACCAAGGCGCTTCCGGAAGCTCAAGAAGG
GACACCACGGTTTGTGGTCTACGACGGGGCACACATTGGCAGCGCTGACTACACGACCGCCGTG
GTGAGTGGAG

MRVPIVKWLPKYSLLDLHGDFVAGMTVALTVIPQGLALADLAKLPIEYGLYTAFMGGFMYAIFG
SCKDLTIGPTAIMSIMTAEYVKHGGPTYAVILTFLSGIIQILMGVLNLGFIVEFISGPVISGFT
SAAAITIASTQLEVSIFSLENSDFVPPSISDFSFILMCRLGDSLLGVSSVILLLVVRHFKDCKF
SQDSRLPPKVRKVIETAWWTIATARNAIVVLACAILASCLLNIGMEPFDLTKEVQGGLPSFRVP
DFSANFNGTNSTTIHKDFFDIVQELGSGIPIIALLSILESVAIAKAFAKGKTLDSTQEMMAIGI
CNLMGSFVSAYPGTGSFSRTAINNNSGVRTPMGGVFTGTIVIMALVFMAPYFKFIPKASLAAII
ITAVIFMIHYQDVPGMWRTNKIDLFPFTATFLVSFVLGLEYGIIAGVVISLALLMYEHARPRIR
ISRKTTLSGVPYLLVSPDRSVLYPSSMYTSSKITKALPEAQEGTPRFVVYDGAHIGSADYTTAV
VSG

>912 (1488 bp)
ATGGGATTCCTCGGATTTGTGAACGTCTACGCCTTACGCGTGAACCTAAGCGTGGCACTCGTGG
CCATGGTCAATCACACCGCCATCTCAACAAACCACACTCCCACGTATGCCCAAGAGTGTCGCCC
GGACGGCTTCAACGAGACCACCGAGAAGCTGCAAGATGGTACGTTCATCTGGAACGAGTACCAG
CAGGGCATTGCACTGGGAGCATTCTTCTATGGCTACGTGCTCACCCAAATCCCGGGTGGGCGCC
TGGCGGAGCGCATTGGTGCCAAGTGGCTCTACGGAGTTGGGGTCTTGATTACTGCGCTGCTTAC
GCTGCTGACTCCCGTGGCTGCCATGTGGAGCTTCTACGCCTTCGTGGCGCTCCGAGTCATGATG
GGGCTGGGAGAGGGCGTGACCTTCCCGGCGATGCACGCCATGATAGCTCGGTGGCTTCCGAAGG
ACGAGCGAAGCTTCCTGTCGACAGTCATCTACTCGGGAGGTCAGATAGGCACCGTCATCGCCAT
GCCCATCTCGGGCATCCTCTGCGACTCCACTTTCCTCGGCGGCTGGCCCGCCGCCTTCTATGTT
TTCGGACTCATTGGCATAGTGTGGTTTGTGTTCTGGGCCCTCCTGGTGTACAATAGCCCCCAGG
AGCACCCCAGGATCAGCGATGAGGAGCGCATCTACATCGAGACCAACCAGGGGGAGGAACAGGC
CAGGGAGAAACTGCCGATTCCCTGGCGAGCGGTGCTGACCTCCCTGCCCTTCTGGGCACTCATG
CTGACCCATTTCGGTCAGAACTGGGGCTTCTACACTCTGCTCACTGAACTACCCAGCTACCTCA
AGAACATCCTCCACTTTGACATCAAGCAGAACGGCTACGTTTCGGCGCTCCCTTACCTGCTGGG
CACCGTGACGAGTTGGAGCGCCGGTATTCTCGCCGACCACATTCGCCGAAGGGACCTCTTCTCG
ACCAGCATCATACGCAAGTTCTTCAACTCTCGTTTTTTCGGGACGGCTGTCTGCCTGTTTGCGG
TGACCTTTGTGGGCTGCAACTACATCCTCAGTGTGGCCTTCCTCACGGTGGGGATGGCCTAAA
CGCCTTCGCCTTCTCCGGGTACATGGTCACCCACGTCGACATGTCTCCGGACTTCGCAGGTACT
TTGATGGGAATGACCAATGCTTTTGCCAACTTGGCGGGATTCCTGGCTCCCCTGGCTGTGGGAA
GCTTGACCAACAACAACGAGACCATCACCCAGTGGAGCATAGTGTTCTACATCGCCTCAGGGAC
GTACATCATCACGGGCGCCATCTTCGTGCTGTTCGGATCGGCGGAGCTTCAACCCTGGGGTCTG
TATGCACAGACGCGCGGCACAAACTTCGCGGGAGCGCTCGGAACTCTCGGAGCGGACCCCTCCG
GAACCGTGCCTTATCAGGCGACTGACGACGGCACTCCTGAAAGTCCCAAGTCTGACGATGCATT
CCCCACGGAAGCATAT

MGFLGFVNVYALRVNLSVALVAMVNHTAISTNHTPTYAQECRPDGFNETTEKLQDGTFIWNEYQ
QGIALGAFFYGYVLTQIPGGRLAERIGAKWLYGVGVLITALLTLLTPVAAMWSFYAFVALRVMM
GLGEGVTFPAMHAMIARWLPKDERSFLSTVIYSGGQIGTVIAMPISGILCDSTFLGGWPAAFYV
FGLIGIVWFVFWALLVYNSPQEHPRISDEERIYIETNQGEEQAREKLPIPWRAVLTSLPFWALM
LTHFGQNWGFYTLLTELPSYLKNILHFDIKQNGYVSALPYLLGTVTSWSAGILADHIRRRDLFS
TSIIRKFFNSRFFGTAVCLFAVTFVGCNYILSVAFLTVGMGLNAFAFSGYMVTHVDMSPDFAGT
LMGMTNAFANLAGFLAPLAVGSLTNNNETITQWSIVFYIASGTYIITGAIFVLFGSAELQPWGL
YAQTRGTNFAGALGTLGADPSGTVPYQATDDGTPESPKSDDAFPTEAY

>432 (1323 bp)
ATGGTGGCCGTGGGATGTTCGTGCATGAACTTCTTCACGGTGGTGATGATTCGGTCGGCGGGCG
TCGTCTACGTCAACATCGTAGACTACTTTGGAGTCACGCGGCAAGAGGCAGCATGGCCCATCTC
CATCGTGCCCGGGTGTGCCAACCTGGCGGGTCCCTTCGTTGCCGTGATCGTCAGAAAAGTCGGC
GTCCGCCCGGTGGCTATCATCGGCACCTTTATGATGTCGCTGTCGATCATGCTCTGCTTTTTTG
CTCCCAACATCCTGTACCTTTCGGTGTTCCTGGGAGTCTTCCATGGTATAGGCTCCGGGATGAC
CATCACACCCAACGCCGTCTGCCTCAACGAATGGTTCGACCGCATGAAGGTCCGAGCGAGCGGC
ATCATATACACGGGAGCCTGTCTGGGTTCGTTTGTGTTTCCCGTGCTTTTCAAGTACTGCAACG
ACGTCTTCGGCATCCGAGGATGCTTCCTCATTTTCGGGGCCCTCATGATGAACGCCGTGGCCTT
TTCCTTCTTCATCCGCTCCCCTCCATGGAGGGTCAAGGCAAGAAAAGCCAAGCTCAAAGCCAAG
CGGATGGAGAAGCAAAAACAGATATTGTCCAACGGAGGCCCCCAGGAAAACAAGGGATTCGTGC
CAGATCTCAAGCTCTCGGCAGGACCGTCCACTGGCGCCATGGTTCCTGTTATACCACAAAGTAA
TGGCTCGTCGAAGGATATCAACATGCAGCCAACCGCACACAGCAACGCCATCAATACCATTCCG
GAACTAGACGACATCGAGGAAGATCATGTATCGATTGCAACGTACCAGCACGTCGAAGTCGCAA
AGAAGAAGCTTGTGAACCGGGTGTCCTTGTTAATGGGGCTCACTTACGTGTTCGTGACCTACAG
CAACATGGCTTTCATGACGGTGCTAATGGACTTCGCGCAAGACAGACACGTGCCACTGGACAAA
GCTGTCTATTTGCTTTCGGGCTTCGCGACTTCGGACATTGTGGGACGATTGAGTGTCGGCTGGG
TGTCTGACAAGGGACTCCTCGAGAACAAGACCATCCTAGGACTGTCTTGCTCGGCTTTCGGCTT
GTGCATGCAGGTTCTGCCATTTTTTTCTACATACGAAGGCATCATGTTCATGTCCATCCTAGTG
GGCTACGCAATCGGCAACACCGTGGTGCTCTTCAATGTCGTCCTCGGTGACGCTGTCGGCGTCG
AGCGCATTCCAACCGCCATCGGCTGTATATCCTTTGTGGCGGGTCTCACTGCATTCTCAAGGCC
CTTTCTGATAGGTGAGCATCTCATTGGGCACCGCGGGTGCTCG

MVAVGCSCMNFFTVVMIRSAGVVYVNIVDYFGVTRQEAAWPISIVPGCANLAGPFVAVIVRKVG
VRPVAIIGTFMMSLSIMLCFFAPNILYLSVFLGVFHGIGSGMTITPNAVCLNEWFDRMKVRASG
IIYTGACLGSFVFPVLFKYCNDVFGIRGCFLIFGALMMNAVAFSFFIRSPPWRVKARKAKLKAK
RMEKQKQILSNGGPQENKGFVPDLKLSAGPSTGAMVPVIPQSNGSSKDINMQPTAHSNAINTIP
ELDDIEEDHVSIATYQHVEVAKKKLVNRVSLLMGLTYVFVTYSNMAFMTVLMDFAQDRHVPLDK
AVYLLSGFATSDIVGRLSVGWVSDKGLLENKTILGLSCSAFGLCMQVLPFFSTYEGIMFMSILV
GYAIGNTVVLFNVVLGDAVGVERIPTAIGCISFVAGLTAFSRPFLIGEHLIGHRGCS

>098 (972 bp)
ATGGCCGTTTACGGATTCATGGCTGCCGATTACATTGTGCTGATCGGATTCTTGGCACTGTCCA
CGTCCATCGGTGTCTTCTTCGCCTGGTCGGATAGACGGCAACAGTCAAACAAGACATTTCTGAC
GGCCAACAAGCAGCTTGGATGGGTTCCCGTCTCCCTGTCAATGATGGCGAGTTTCCTGTCGTCC
ATCGCCATCCTGGGCTTGCCTTCGGAAGTGTTCGTTCACGGCTCTTCATTGTGGATGGGTGCCG
TTTCTTCGACCATTGCTGTCATCTTAGCAGCGTACGTGTTCCTGCCCATGTACTACAAGATGGA
CATCACTAGCATCAACGAGTACCTGGAACGGAGGTTCAATTCAACAGCGGTCAGAAACGTGGCT
TCCGGCGTCTTCATCGTTCAAACGCTGCTGTACATGGGAGTGGTGCTGTACGGTCCATCATTGG
CGCTTGGCTCTGTGACTGGAATTCCCGTCTGGATGACCATCGTCCTCAACGGACTGGTGTGCAC
CTTCTACACAGCCATTGGCGGAATCAAGGCTGTTGTATGGACCGATGTGATACAGATGATCCTT
ATCTACGTAGGCTATATAATGGTAATCATATCGGGATTGCATCACCTTGGGGGCTTCGGCAACA
TGTGGAAGCTTGCTGAAGAAGGTGGACGAATCATCGTTTTCAATTTCAGCTTCAGTGCGTACGA
GACCTACACGACGTGGAACGTGATTCTTTCTTGGACCATCGGGTGGATGTCTGCCTACTGCGCC
AGCCAGACGCAGGTCCAGCGGTACTCGAGCATCGCCTCACTCAGAGACGCCAGGAGAGCGCTGC
TTCTGAACGTGCCAGGAGTGGCGTTGACGCTCCTCCTCGCCGTACTCTCGGGTCTGATCATCTT
CGCCATATACAAAGACTGTGATCCTCGACTGCTCGGGGAAATCGACAAGGCGGATCAGGTGCGT

GCTCTCCCGATT

MAVYGFMAADYIVLIGFLALSTSIGVFFAWSDRRQQSNKTFLTANKQLGWVPVSLSMMASFLSS
IAILGLPSEVFVHGSSLWMGAVSSTIAVILAAYVFLPMYYKMDITSINEYLERRFNSTAVRNVA
SGVFIVQTLLYMGVVLYGPSLALGSVTGIPVWMTIVLNGLVCTFYTAIGGIKAVVWTDVIQMIL
IYVGYIMVIISGLHHLGGFGNMWKLAEEGGRIIVFNFSFSAYETYTTWNVILSWTIGWMSAYCA
SQTQVQRYSSIASLRDARRALLLNVPGVALTLLLAVLSGLIIFAIYKDCDPRLLGEIDKADQVR
ALPI

>624 (1164 bp)
ATGTACCTGGAACTCCGATTCAACCACATCATTCGTACCATTGGGTGCCTCACCTTTTCAATTC
AGATGTTAATTTATATGGCCATCGTCCTGTACGCTCCTGCGCTGGCACTTTCCCAAGTGACTGG
AATCAGTGTGTGGACGTCGGTGCTCTCCATAGGCATCGTGTGCACGTTCTACACAAGCGTGGGC
GGCATCAAGGCGGTGGTGTGGACGGACGTGTTCCAGATCTTCCTCATGTTCTCGTCCATGCTGC
TCGTGGCCTTCAAAGGGGCCTACAGCATTGGTGGCTTCGGCTACGTCTTTGAACTGGCCAGCAA
AAACCAGCGGGTCGAGTTCTTCAATTTTAATCCAGACCCCACGGACCGCCACACAGTCTGGGGT
CTAATCATCGGTTGCTACTTTACGTGGATGTCTATTTACGCTGTGAGTCAAGCCATGGTTCAGC
GGTACCTGACCCTCCCGAACCTCAGAGGAGCCAGACAGGCAATCTGGTTCAACCTGCCTGGCCT
TTCGTTCCTGTTAATTATATGCGCTTTGGCCGGGCTCGTTATGTATGCCAAGTATTACGACTGC
GATCCCCTGCTCACGAAGAAAGTCAGCTCAAGTGATCAGCTTCTGCCTTTGTACGTAATGGATA
TTCTTGGACTATTTCCCGGGATCCCAGGACTGTTCGTTTCTGGCATCTTCAGCGGCGCTTTAAG
TACCGTATCTTCGGGCGTGAATTCTCTAGCCGCCGTCACGCTGGAAGACGTCATAAAGAGACAC
ATCAAGAGCGACATGTCGGATCGCTTCGCTACAAACTTGACGAAGGGCCTAGCAATGTCGTACG
GATTCATCGCCATCATACTTGTAATCATTGCACAAAACTTGGGCAACGTACTGCAGGCAGCGTT
GTCAATATTTGGGATCGTCGGAGGACCCCTTCTGGGCGTCTTCACCCTTGGAATATTCTTCCCT
TTTGCGAACAGCATTGGTGCAGGCGTCGGCACCATTGGCTCGCTGATTATTTGTTTCTGGATTG
GCTTCGGCTCCTTCTACTTCAAGCCAGCCGTTCGCAAGCCGCCCGTTTCCGTCCTCGGCTGCCT
ACCACTATACCTTAACGTGACTGGACAACGTGGCAACGTCACCCTCCCTGTTCCTGTGGACGTA
GATCTGGCAAAG

MYLELRFNHIIRTIGCLTFSIQMLIYMAIVLYAPALALSQVTGISVWTSVLSIGIVCTFYTSVG
GIKAVVWTDVFQIFLMFSSMLLVAFKGAYSIGGFGYVFELASKNQRVEFFNFNPDPTDRHTVWG
LIIGCYFTWMSIYAVSQAMVQRYLTLPNLRGARQAIWFNLPGLSFLLIICALAGLVMYAKYYDC
DPLLTKKVSSSDQLLPLYVMDILGLFPGIPGLFVSGIFSGALSTVSSGVNSLAAVTLEDVIKRH
IKSDMSDRFATNLTKGLAMSYGFIAIILVIIAQNLGNVLQAALSIFGIVGGPLLGVFTLGIFFP
FANSIGAGVGTIGSLIICFWIGFGSFYFKPAVRKPPVSVLGCLPLYLNVTGQRGNVTLPVPVDV
DLAK

D. reticulatus

>S1 (303 bp)
ATGGATGGAGATGGGCCATTCACAGTGTTTGGTCCAGTGGATGAGGCATTCGCAAAACTGAGTC
CGGAGGTAATTGACCATCTCAAGAGCAACGTTACAGCCCTGAAAGAGGTTCTGCTTTACCACGT
GGTCCCCGACGTATGGTTCAGTGCCGGGCTGGTGAATGGCCAGCTGAAGACTGTGCAGGGACAG
AACCTGACTATTTCCATCGATGACGGTGGTGTTCACGTCAACGATGCTACCGTCAACTTGGCCG
ACGCAGCCGTGAGCAACGGCGTAGTCCATTCGATTGACACCGTCCTC

MDGDGPFTVFGPVDEAFAKLSPEVIDHLKSNVTALKEVLLYHVVPDVWFSAGLVNGQLKTVQGQ
NLTISIDDGGVHVNDATVNLADAAVSNGVVHSIDTVL

>S2 (363 bp)
ATGACGCTAAGTGTATTAACAGCGCTAACTTTTTGCTGCTTCTTGTCCACGGCATTTCACAGAT
ACCAGTCGGAAGTTGAACTGCAAGCCATCAAAGCTGTTGTGAAGAACGTCCAGGACTTCAGTAC
AGCAAGAGGAAAGAATGACCTGGGCTTTGTCACGTTCGACCTTAAAGCCACACTTAATCAAGTT
GTCTTATGGGATAAGATAATCAGGCGGGGAGATAATGCCATTTTGGACTACAAGAACCTAAACA

CAAAGTACTACTTCTGGGATGATGGCCATGGACTGAAGGGCAACAGGAATGTAACGCTCACCCT
GTCCTGGAACGTCATCCCCAATGCAGGCAGCCTGCCCAACATC

MTLSVLTALTFCCFLSTAFHRYQSEVELQAIKAVVKNVQDFSTARGKNDLGFVTFDLKATLNQV
VLWDKIIRRGDNAILDYKNLNTKYYFWDDGHGLKGNRNVTLTLSWNVIPNAGSLPNI

>S5 (657 bp)
ATGGGCGAAGCAAAGAACCTGCCGCCTAGAAGCCATGGCTCGGTGGAACAGCGTGACGTCTACT
GCGCCATCAAGCTCGACCAGGAGGAGATATTTCGCACGACGACCAAGGAGAAAACCCTCAATCC
CTTCTTCTCCGAGGACTTCCAATTTGAAGTGCCGCGGGAGTTCCGGTACCTCTCGGTCTATGTG
GCTGAGCGAGAGCGGGCCTCTAACAAGGAAACCTTCAAGCGTCGCTACTTCTGCCTCACCACCA
AGGAGCTGTACTACTCAAAGACGAAAGACTCGAGTCCACTGTGTGCCATTCCACTGGATGAAAT
CTTGGGAGTGGAAAAGGTGCAGGAAGACTCATTCAAGATGAAGAATGTGTTTCAGGTGATCCAG
AAGTCCCGAGCGTTGTACATCCAGGCCAACAACTGTGTAGAAGAGAAGGAATGGCGAACAATCC
TCACCCGCATCTGCCACTTCAACTTGTGCCGCATCCACACCTACCATCCGGCAGCCTTTCTCAA
GGGCCACTGGCTCTGCTGCAAAGAGGAGAGTGAAACGGCGCCTGGCTGCTCGCCCGTGACCTCG
TATAACCTCGCTGACATCAAGGTGACCATCGACACAGACCGAGAGATGCAGCGGGTCCACTCTA
TTTTCCTCAACCAGATG

MGEAKNLPPRSHGSVEQRDVYCAIKLDQEEIFRTTTKEKTLNPFFSEDFQFEVPREFRYLSVYV
AERERASNKETFKRRYFCLTTKELYYSKTKDSSPLCAIPLDEILGVEKVQEDSFKMKNVFQVIQ
KSRALYIQANNCVEEKEWRTILTRICHFNLCRIHTYHPAAFLKGHWLCCKEESETAPGCSPVTS
YNLADIKVTIDTDREMQRVHSIFLNQM

>S6 (297 bp)
ATGGGATTCTTGGGCTTTGTCAACGTGTATGCACTAAGAGTGAACTTGAGTGTTGCCCTGGTTG
CCATGGTTAACCACACCGCAATCCTTGCCAACAGCACGCCTTCATCAGGAGTGATGGTGACCCA
CTTTGGCCAGAACTGGGGCTTCTACACGCTTCTCACCGAAATGCCTAGTTATCTCAAGAACATC
TTGCACTTTGACATCAAGAAGAACGGGTACCTCTCAGCACTGCCGTACCTCTTGGGGACATTCA
CCAGCTGGGGAGCCGGATATCTGGCTGACCACATACGGCGA

MGFLGFVNVYALRVNLSVALVAMVNHTAILANSTPSSGVMVTHFGQNWGFYTLLTEMPSYLKNI
LHFDIKKNGYLSALPYLLGTFTSWGAGYLADHIRR

>S7 (240 bp)
ATGTTGGACAGCGTAAAGATCAAGAACGCCGTGGTGCGTGAAGTCCTCGCTGAACTCCTGGGAA
CGTTCGTCCTTGTGGCGCGAGACCTGGCGCCCAGGGTTTTCACTGCCATGGCCGGATGGGGAGT
TGAGGTTTTCAGCTACCGGGATTACAACTGGTTCTGGGTGCCCATCGTGGGCCCGCACATCGGC
GCCATCGTGGGCGCCTGGTTGTACATGCTGGCTGTGGAGCTTCACTGG

MLDSVKIKNAVVREVLAELLGTFVLVARDLAPRVFTAMAGWGVEVFSYRDYNWFWVPIVGPHIG
AIVGAWLYMLAVELHW

>S8 (300 bp)
ATGGTGTACTTCCACACGGTGAACCCGCGGCTGGCCGACTTCAGCGACGACTACAAGGCCTGCC
TCAAGGGGGCGCAAGGAAGGCTCAGACCCTTCGGAGACGCCCCGCTTAGGCTCAAGGGTTCCTT
GGCCGAGTCCCTAGGCGCTGTTCGCACTGTACTTCGGGCAACGAGACTGGGTGCTTACAGCCTG
GACGAGGCCTTGTCACTGCTGGACTCAAGGGTATCCGAGGCCGTCATCCACGCCATGGAGAATG
GTCCTGAACTTTCAAAGAGGGTGAAGTTAGAATGTGGTGACCCT

MVYFHTVNPRLADFSDDYKACLKGAQGRLRPFGDAPLRLKGSLAESLGAVRTVLRATRLGAYSL
DEALSLLDSRVSEAVIHAMENGPELSKRVKLECGDP

>S9 (171 bp)
ATGGATTTCGAGGGACAGAAGGCAGCGGAGAAAATATTTCAAGTGGTCATAGTTGCCTTTGCGG
CGGCTGGACTTGTCTGGGGGTACATTGTACAGCAGTTCTCCTACACCGTCATCAGCCTCGGAAT
TGGTTTTGTCATTTCCTGCCTGCTCACACTGCCTCCCTGGCCA

MDFEGQKAAEKIFQVVIVAFAAAGLVWGYIVQQFSYTVISLGIGFVISCLLTLPPWP

>S10 (429 bp)
ATGCATCTCCACGGCGCTCTCGAACAACCTTTTGCCGCCAAGTTCGACGAAGGAAAGATCGAGG
AGATCGAGATCGGCAAGAGCGAACCTCTGTGGGTCAGGAACGTGAAGAAGGGCGTGCTGTCCCT
CTTCCAACTGGACCTCGTCAAGGGACGTCACGAGCACCACGATGACGACACGCTCACCCTTGAG
TTCGAGAGCTGGGGCCTCGACAAGTTCCTCAATCACCTCCTGGGCCCACAGCCCGGATCCACGA
AGAACCTTTGGAATTTCATGGGCCGTCGCCGCATCCCGCGTGATGCATCGGCTAAGGAGCGAAA
GGAGATCGATGAAGCCCTGCACATCTCCGACCGAGAGTACGACCCAATGTACGCACGTCTTAGC
CTGTCCATCTTTGGAAAGACCGTCGATACTTATAGCATCGACGAG

MHLHGALEQPFAAKFDEGKIEEIEIGKSEPLWVRNVKKGVLSLFQLDLVKGRHEHHDDDTLTLE
FESWGLDKFLNHLLGPQPGSTKNLWNFMGRRRIPRDASAKERKEIDEALHISDREYDPMYARLS
LSIFGKTVDTYSIDE

>S11 (489 bp)
ATGCTTCCACACAAAGATGCAGTGTGGGCTGACCAGGCCATCAAGTCGGAGTTCTTGTCAGTGG
AGAAGCTTCTCATTCAGACGATCCACATTCGAACGAAACAGCGTCTGTCTGATCTCCGAGACCG
GCTCAAAAGTTTTCTTGGGTCCTCCGTAGAATGTCCAATCCAGGGCTCGCCAGCCGTGCTGCAG
GTTCCCCTTCTGCAGCCCTGCATGCAGTCGGAGAACCTGCTTGTCACTGTGGACACCCATACAG
GATACTTCCTGGCGTTTGTACCTCAGTACAATCCTCCCATGATTGGTGACATTCAAGAGGCACT
TAACAAAGATACCAGCAAGCTTGACAGCCTCCTGATTGATCTCAAGTTTTGGATGACGGTGAAA
CGTTGCGAAAAGACACTGCAGCATCTGCCTGTTTTGACGACCACAACGATCCCCTTGGTGGTTC
CTCGGGGCCACATTGCCAGCAAGCTGGGCCCGCACACCCTC

MLPHKDAVWADQAIKSEFLSVEKLLIQTIHIRTKQRLSDLRDRLKSFLGSSVECPIQGSPAVLQ
VPLLQPCMQSENLLVTVDTHTGYFLAFVPQYNPPMIGDIQEALNKDTSKLDSLLIDLKFWMTVK
RCEKTLQHLPVLTTTTIPLVVPRGHIASKLGPHTL

>S12 (759 bp)
ATGATGGCCAGCTTCCTCTCTTCCATCGCTATCCTCGGACTTCCGTCGGAGGTGTTCTTGCGCG
GTTCGACCATCTGGACGGGGGCTATATCTTCAAGCCTGGCTGTCCTCGTGGCGGCTTTCGTCTT
CCTGCCCATGTACTACAAAATGGATATCACCAGCATCAACGAGTATTTGGAGAAGAGGTTCATG
TCTACTGCTGTAAGGAACATCGCGTCAGCCGTCTTTATTGTGCAAACGCTCCTTTACATGGGCG
TTGTGCTCTACGGCCCTTCGTTGGCTCTTGGATCTGTGACGGGCATACCGGTATGGTCCTCCAT
TCTCCTGAACGGCGTCGTATGCACCTTCTACACCGCCATAGGAGGAATCAAAGCGGTCGTGTGG
ACGGACGTGGTCCAGATGATTCTCATTTACGTCGGCTACATTATGGTCATCGCATCGGGCATGT
ACCACTTGGGAGGCATCGGCAACGTGTGGCACATCGCCGGCGAAGGAGGTCGGCTTGTTTTCTT
CAACTTCAGCCCGAGCCTTTACGACACGTACACCACGTGGAACGTCATACTCGGCTGGACCATC
GGTTGGATGGCCGCCTACTGCGCCAGCCAGACGCAGGTGCAGCGCTACTCAAGCATGAAGTCGC
TCAAACGAGCACGCAGGGCGCTGCTGCTCAATATCCCCGGCGTGGCCTTGACCCTGCTGCTATC
CGTGCTGTCGGGGCTAACCATCTACGCCGTGTACCGGGACTGCGACCCGAGGCTC

MMASFLSSIAILGLPSEVFLRGSTIWTGAISSSLAVLVAAFVFLPMYYKMDITSINEYLEKRFM
STAVRNIASAVFIVQTLLYMGVVLYGPSLALGSVTGIPVWSSILLNGVVCTFYTAIGGIKAVVW
TDVVQMILIYVGYIMVIASGMYHLGGIGNVWHIAGEGGRLVFFNFSPSLYDTYTTWNVILGWTI
GWMAAYCASQTQVQRYSSMKSLKRARRALLLNIPGVALTLLLSVLSGLTIYAVYRDCDPRL

>S13 342 bp)
ATGCACTGGATACCGGTTGGTGCTTCGCTTTTCGCCAGCAACATCGGAAGCGGCCACTTTGTTG
GCCTCGCTGGATCTGGCGCCGCTACGGGCATTGGAATCGCGTCGCTGACGTCCATCTTCAACAG

CTCTTCGACCATCTTCACCATCGACATCTGGAAAAAGTTCCGCAAGCAGGCCTCCGACGTGGAG
CTGCTCATTGTGGGCCGTTCCTTCGTCGTCGTCCTCGTTGCGCTGAGTATCGTGTGGATCCCCA
TCATCGAGCACTTCCCCAGCAGCCAACTCTTCCACTACATCCAGAGCGTCACCAGCTACCTGGC
GCCGCCCGTCTGCGCCGTCTAC

MHWIPVGASLFASNIGSGHFVGLAGSGAATGIGIASLTSIFNSSSTIFTIDIWKKFRKQASDVE
LLIVGRSFVVVLVALSIVWIPIIEHFPSSQLFHYIQSVTSYLAPPVCAVY

>S14 (309 bp)
ATGAGGACCTTCGCCCTTTTCGCTGCGGTTTTCGCCTTCGCCGCCTACCAGGTGAACGGTGAGG
CCTGCAACTGCCACCTGCGCGAGCTGGATCTCTGCGCGGCGACGCTGCTGCTCTTCAACCAGAA
CCCATCCGGAGTGGCCACCACCGATGCTGAGGTCGACAAGCAGTGCGGCTTCCTCAAGGAGTCC
CAGGACTGCTTCAGGAACTTCACCACTCGCTGCGCCACTCCCCTGCAGAGGGAACTGATCGGCT
TCGTCGCTGAGGGATCCCAGGAGCTCTTCAAGCAGTTCTGTACCAAGGGAACC

MRTFALFAAVFAFAAYQVNGEACNCHLRELDLCAATLLLFNQNPSGVATTDAEVDKQCGFLKES
QDCFRNFTTRCATPLQRELIGFVAEGSQELFKQFCTKGT

>S15 (834 bp)
ATGGAAGAAGGACCACCCCATACTAAGAACTGGAGACCCCAAGTGCTTGTCCTGTGCAAACTCA
ACCAAGACTACATGCCCAAATATCGCAAGCTTATCACCTTTGCCTCACAACTCAAGGCTGGAAA
AGGCCTGACACTTGTCTGCTCTGTCTTGGAAGGAGAGTACAGCAAAATGTACAGTGAATGCCAA
GCAAGCAAACAGAGCTTGAAGAAAGTACTTGAAGAAGAGAGAGTGAAGGGCTTTGCGGATGTCG
TTTCTGGAGGAAACACCATTGATGCCATCTGCCATATCATCCAAACTGCTGGCCTTGGTGGACT
CAAACACAACACTGTTATCCTTGGCTGGCCGTATGGATGGAGGCAGTCTCCTGATGAGCGCTCT
TGGAAGGTTTTCATTGAAACTATTCGAAACGTTTCGGCAAGCAAGAATGCCCTCCTTGTGCCAA
AGAACATCAACCAGTTTCCAGATAACACCGAGAAGCTCCATGGAACCATTGACGTGTGGTGGAT
TGTTCATGATGGTGGCCTGCTCATGCTCTTGCCATTCCTGCTGAAGCAGCACAAAGTTTGGAAG
AACTGCAAGCTGAGGATCTTCACTGTTGCTCAGCTTGAAGACAACAGCATTCAAATGAAGAAAG
ACCTGGCCATGTTCCTCTACCACCTGAGAATAGATGCAGAAGTGGAAGTTGTGGAAATGAATGA
CAGTGACATCTCGGCGTACACCTATGAAAGAACACTAATGATGGAGCAGCGTACAGAGATGCTC
AAGCATATGAGGCTGAGCAGGCGAGAAACCTTGAGCATGATTCAAACAATTGTGGATCATCACC
AC

MEEGPPHTKNWRPQVLVLCKLNQDYMPKYRKLITFASQLKAGKGLTLVCSVLEGEYSKMYSECQ
ASKQSLKKVLEEERVKGFADVVSGGNTIDAICHIIQTAGLGGLKHNTVILGWPYGWRQSPDERS
WKVFIETIRNVSASKNALLVPKNINQFPDNTEKLHGTIDVWWIVHDGGLLMLLPFLLKQHKVWK
NCKLRIFTVAQLEDNSIQMKKDLAMFLYHLRIDAEVEVVEMNDSDISAYTYERTLMMEQRTEML
KHMRLSRRETLSMIQTIVDHHH

>S16 (690 bp)
ATGTATCTGGAGCTCCGTTTCAATCACGTCATTCGAGCCATGGGATGTATAACGTTCTCCTTAC
AAATGCTCATCTATATGGCGATTGTTCTTTACGCACCGGCACTTGCACTTTCCCAAGTGACGGG
AATCAGTGTATGGACCTCGGTACTTTCCATAGGCATTGTCTGCACTTTCTACACAAGCATTGGC
GGAATGAAAGCTGTCGTGTGGACGGACGTGTTCCAAATATGCCTTATGTTTGGCTCCATGCTCA
TGATCGCCATCCGCGGAGCGCACGACATCGGTGGTATGAAGGTCGTCTACAACAGGGCCTCCGA
CGGAAACAGAGTCGAGTTCTTCAACTTCAGCCTCGACCCGACCGAGAGGCACACCGTCTGGGGT
CTCTTCATCGGCTGTTTCTTCACTTGGATGTCGGTGTACGCAGTCAGCCAAGCAATGGTCCAGC
GATACCTCACAGTTTCCAGCATGAAAGGGGCTCGAATCGCCATCTGGATCAACCTTCCTGGACT
GGCGTTTTTGATGTTGATCTGCGCTCTGGCTGGCCTCGTCATGTACGCCCGGTACCAAGATTGC
GACCCCCTGCTGACCAAGAAAGCGACATCCCCTGACCAGCTACTTCCTCTCTACGTCATGGACA
TCCTCGGAAGCCTGCACGGAATTCCTGGCCTATTTGTCTCCGGCATATTC

MYLELRFNHVIRAMGCITFSLQMLIYMAIVLYAPALALSQVTGISVWTSVLSIGIVCTFYTSIG
GMKAVVWTDVFQICLMFGSMLMIAIRGAHDIGGMKVVYNRASDGNRVEFFNFSLDPTERHTVWG

LFIGCFFTWMSVYAVSQAMVQRYLTVSSMKGARIAIWINLPGLAFLMLICALAGLVMYARYQDC
DPLLTKKATSPDQLLPLYVMDILGSLHGIPGLFVSGIF

>S17 (663 bp)
ATGCAAACCAATGAGGAAATACCAGGGAAAGGCATAATTCTCGACACAAGATCGCATACCCTAG
CTTTACAGTGCAACTACACCGATGACCCCAGCAAGGAAATCCTTTGGTACAAGGATGGTGCCAA
GCTTTCGAGTGAAGATAAGAAGTACAGCATCAACAGCGATTCCCACACCCTGGAGGTCAATGAT
CCAGGATACCCAGACACTGGAAACTACACATGCGTTGTTGTTGGCACTGACGAGAATGCCACAA
TTGTTGTTCAGACCAACGTGTCCATTGAGTATGCTGAAAGCTCCAAGAACCAAGTGGAAGGAGA
CCCACTCACCCTGTCTGTCACTCTGGAGCCGATCAACAATGTGACCGATGCCAAGCTTGTAATT
CAGGACCTCAACTTCGATGACAGGGCCCAGTACACTTGCGTTGCCAGCAACGGTATCAGCAATG
AGACCATGACAGTTTTGGTTCGCGTCAAGGACAAGTTGGCTGCTCTGTGGCCCTTCCTTGGCAT
CTGTGTTGAGGTGGCCGTGCTATGCGCCATTATCTTTATCTATGAAAAGAAGCGTGTCAAGCCC
GACTTCGAGGAGTCTGACACTGACCAGAATCCTGAAAACAAAAACCTGTCAGATCAGAAGGAAG
GCCAGGACATACGACAAAGGAAG

MQTNEEIPGKGIILDTRSHTLALQCNYTDDPSKEILWYKDGAKLSSEDKKYSINSDSHTLEVND
PGYPDTGNYTCVVVGTDENATIVVQTNVSIEYAESSKNQVEGDPLTLSVTLEPINNVTDAKLVI
QDLNFDDRAQYTCVASNGISNETMTVLVRVKDKLAALWPFLGICVEVAVLCAIIFIYEKKRVKP
DFEESDTDQNPENKNLSDQKEGQDIRQRK

>S18 (525 bp)
ATGGCTCTCACTGTCATCCCACAGGGCCTGGCACTGGCTGTTCTTGCCGGCCTACCTCCACAGT
ATGGGCTGTACACGGCATTCATGGGAAGCTTCATGTACACGATCTTTGGGAGCTGCAAGGACCT
CACCATTGGCCCCACAGCCATCATGTCCATCATGACTGGGGAGTACACACACGTTGGCGGGCCC
ACGTTTGCCATCATCCTCACATTCCTCTCGGGAGTCATCCAGATTCTCATGGGATTGCTCAACC
TAGGTTTCATAGTGGAATTCATATCTGGCCCTGTAATCAGTGGTTTCACGTCAGCAGCTGCCAT
CACTATTGCAAGTACACAACTCAAGTCTCTATTTGGAATGAAGTTTGAGGCTGAGGAGTTTCTC
GACACCATGTACCAGTTCTTCACGCACCTTTACACCATGAGGTTGGCAGATTCCTTGCTGGGTG
TCACGTGTGTCATTCTGCTGCTCCTCATCAGGCATTTCAAGGATATGAAGTTCAGCCCGGATTC
CAGGGTTCCGCCT

MALTVIPQGLALAVLAGLPPQYGLYTAFMGSFMYTIFGSCKDLTIGPTAIMSIMTGEYTHVGGP
TFAIILTFLSGVIQILMGLLNLGFIVEFISGPVISGFTSAAAITIASTQLKSLFGMKFEAEEFL
DTMYQFFTHLYTMRLADSLLGVTCVILLLLIRHFKDMKFSPDSRVPP

>S19 (177 bp)
ATGATCTTCGGCATCATCTTGGGCATCGTGCTGTTCGTCATCATCAGCATCTCGCTAATCGGCC
TGTACGTGGCTTTCAAGCGCTTTTGCTCGGACACCAAGGACTATGGCATGATCAACGCCAACTC
GACTATCAAGCACGGTCGCCTGTACCCGGTGCTCAACTACAAGGACTGC

MIFGIILGIVLFVIISISLIGLYVAFKRFCSDTKDYGMINANSTIKHGRLYPVLNYKDC

>S20 (111 bp)
ATGTACGTGGCTTTCAAGCGCTTTTGCTCGGACACCAAGGACTATGGCATGATCAACGCCAACT
CGACTATCAAGCACGGTCGCCTGTACCCGGTGCTCAACTACAAGGACTGC

MYVAFKRFCSDTKDYGMINANSTIKHGRLYPVLNYKDC

>S21 (231) bp
ATGGGAGCAGACGTAGCTGCCGTTTCTATTGTAAGTGTTTTCTGGGTCGTCGTCGGTGCTATCG
TACCATGGTTCATACGGAAGGGCTCGCATCGCAGCCTTATTCAGGCCATGATCGTCACCACATC
AATATGTTGCTACCTTTGGCTGTGCACATACATGTCCCAAATGTACCCCTTGGTTGGTCCGTCG
CTAAGCAACACTACTGTGATCATCATGGGAGAATACTGG

MGADVAAVSIVSVFWVVVGAIVPWFIRKGSHRSLIQAMIVTTSICCYLWLCTYMSQMYPLVGPS
LSNTTVIIMGEYW

>S22 (276 bp)
ATGGTGGGGTGCGATGCCAGGGCGGCGCTGGTGTGCCTGGTTTCTGGCATGGCCCTGTACGGAT
TCACCGTCGGGGGACAGAGCCCACTGACCCTGGACATCGCGCCAGACTTTGCAGGCACGGTGAT
GGGCATTGTGAACTGCATGGGCAACCTGTCGGGCATGCTGGCGCCCCTGGTGACCGGCTACATC
ATTGAGCACGATGAGAGCCTGGCGCAGTGGCGCAAGTTGTTCCTGCTGGCATCGGCCATCTACA
CTTTTGGTGCAGTGAGCTTC

MVGCDARAALVCLVSGMALYGFTVGGQSPLTLDIAPDFAGTVMGIVNCMGNLSGMLAPLVTGYI
IEHDESLAQWRKLFLLASAIYTFGAVSF

>S23 (477 bp)
ATGGAAACGCTGTGGATGGTGTCAGCCGAAAAGGAACGCTACCAGTGCCTGCTGCCCGCCAAGC
CATTATCACGAGAGGATGCCGACGCTGCTTCCCCGTACACGGGGCCAACGCCGCTGGAGCTGCT
GAAGCCGCTCTTTTCCCGGCTCTTCTGTTCCTACCGCCTGGAGCAGTACTGGACCTACGAGCTG
TGCCATGGCAAGAGCGTTCGGCAATACCACGAGGAGAGCATCGACAACAAGGTAGTCCTGCAAC
AGTACTACCTTGGCAAGTACGACACCAAGAAGCTGGAAGTTGATGATGCGAGCTACTTATCGGA
GAGAAGCCGGCGGCCGCCCCGCATTCGGCTGGAAGCGGGCAAGCCTCGCACAGTGGAAGTCCTC
CTGAAGTGCCGCCAGGCGAAGGGCAACCTTGACAGTGTGTCCCTGTACCTGCTGGAGCCACGCA
CCTGCGAGTACATTCTCGGGGTGGAGTCT

METLWMVSAEKERYQCLLPAKPLSREDADAASPYTGPTPLELLKPLFSRLFCSYRLEQYWTYEL
CHGKSVRQYHEESIDNKVVLQQYYLGKYDTKKLEVDDASYLSERSRRPPRIRLEAGKPRTVEVL
LKCRQAKGNLDSVSLYLLEPRTCEYILGVES

>S25 (333 bp)
ATGGACCTCTACAATGCCTTGGATGGAGATGGGCCATTCACAGTGTTTGGTCCAGTGGATGAGG
CATTCGCAAAACTGAGTCCGGAGGTAATTGACCATCTCAAGAGCAACGTTACAGCCCTGAAAGA
GGTTCTGCTTTACCACGTGGTCCCCGACGTATGGTTCAGTGCCGGGCTGGTGAATGGCCAGCTG
AAGACTGTGCAGGGACAGAACCTGACTATTTCCATCGATGACGGTGGTGTTCACGTCAACGATG
CTACCGTCAACTTGGCCGACGCAGCCGTGAGCAACGGCGTAGTCCATTCGATTGACACCGTCCT
CATACCAAAGCTT

MDLYNALDGDGPFTVFGPVDEAFAKLSPEVIDHLKSNVTALKEVLLYHVVPDVWFSAGLVNGQL
KTVQGQNLTISIDDGGVHVNDATVNLADAAVSNGVVHSIDTVLIPKL
