# Supplementary material for: A Vaccinomics Approach for the Identification of Tick Protective Antigens for the Control of Ixodes ricinus and Dermacentor reticulatus Infestations in Companion Animals
Source: Front Physiol. 2019 Jul 26;10:977. doi: 10.3389/fphys.2019.00977 (PMC6681794; doi:10.3389/fphys.2019.00977)
Supplement: Supplementary file 7 [file Table_1.pdf]

Supplementary Table 1. Primers and conditions for gene expression analysis by real-time RT-PCR of *I. ricinus* and *D. reticulatus* tick genes encoding for candidate protective antigens.

I. ricinus gene ID	Forward (FW)/Reverse (RV) primer sequences (5' – 3')	Annealing temperature (ºC)
082	**FW-**ATGCCGAAACAAGGCGAAAC<br>**RV-**TCCAGAGTCACCACACAAAAC	58
391	**FW-**ATGGTTAACTATATATCCTACT<br>**RV-** GGGAGCGGTGCAGTCAGACGCG	64
749	**FW-**ATGATCGCCCAGGCTCC<br>**RV-**CTTCAAGAACGTCACTCCAATC	54
216	**FW-**ATGAGGACCTTCGCCCTTTTC<br>**RV-**GTTGCCCAGGTAGGCGGAG	62
D. reticulatus gene ID	Forward (FW)/Reverse (RV) primer sequences (5' – 3')	Annealing temperature (ºC)
S2	**FW-**ATGACGCTAAGTGTATTA<br>**RV-**GATGTTGGGCAGGCTGCC	54
S8	**FW-**ATGGTGTACTTCCACACG<br>**RV-**AGGGTCACCACATTCTAA	59
S10	**FW-**ATGCATCTCCACGGCGCT<br>**RV-**CTCGTCGATGCTATAAGT	56
S12	**FW-**ATGATGGCCAGCTTCCTC<br>**RV-**GAGCCTCGGGTCGCAGTC	59
S14	**FW-**ATGAGGACCTTCGCCCTT<br>**RV-**GGTTCCCTTGGTACAGAA	59
S16	**FW-**ATGTATCTGGAGCTCCGT<br>**RV-**GAATATGCCGGAGACAAA	54
S17	**FW-**ATGCAAACCAATGAGGAA<br>**RV-**CTTCCTTTGTCGTATGTC	56
S18	**FW-**ATGGCTCTCACTGTCATC<br>AGGCGGAACCCTGGAATC	52
S20	**FW-**ATGTACGTGGCTTTCAAG<br>**RV-**GCAGTCCTTGTAGTTGAG	51
rsp4	**FW**-GGTGAAGAAGATTGTCAAGCAGAG<br>**RV**-TGAAGCCAGCAGGGTAGTTTG	52
